# Association between psychosocial factors and adverse effects of light-to-moderate ambient heat in patients with chronic diseases: results of the prospective cohort study CLIMATE-II

**DOI:** 10.1186/s12916-026-04622-4

**Published:** 2026-01-15

**Authors:** Ingmar Schäfer, Valentina Paucke, Julia Nothacker, Agata Menzel, Susanne Döpfmer, Klaus Hager, Susann Hueber, Arian Karimzadeh, Thomas Kötter, Christin Löffler, Beate S. Müller, Martin Scherer, Dagmar Lühmann

**Affiliations:** 1 Institute and Outpatients Clinic of General Practice/Primary Care, University Medical Center Hamburg-Eppendorf, Hamburg, 20246 Germany; 2 Institute of General Practice, Charité — Universitätsmedizin Berlin, Berlin, Germany; 3 Institute of General Practice and Palliative Care, Hannover Medical School, Hanover, Germany; 4 Institute of General Practice, University Hospital Erlangen, Friedrich-Alexander-Universität Erlangen-Nürnberg, Erlangen, Germany; 5 Institute of Family Medicine and General Practice, University Hospital Bonn, Bonn, Germany; 6 Institute of Family Medicine, University Medical Centre Schleswig-Holstein, Campus Lübeck, Lübeck, Germany; 7 Institute of General Practice, Rostock University Medical Center, Rostock, Germany; 8 Institute of General Practice, University of Cologne, Cologne, Germany

**Keywords:** Climate change, Heat, Ambient temperature, Chronic diseases, Psychosocial factors, Risk expectations

## Abstract

**Background:**

Global temperatures are increasing. Adaptation of health behavior could mitigate adverse effects of heat, but health benefits are probably limited and depend on the context. Therefore, other strategies are also needed. Our study aimed to identify adverse effects of light heat (> 27–32 °C) and moderate heat (> 32–40 °C) and to analyze whether psychosocial factors were associated with these effects.

**Methods:**

We conducted a prospective cohort study based on an access-restricted online survey and publicly available weather data. A total of 1810 individuals were contacted by 64 GP practices in 16 German federal states. Individuals were eligible if they were ≥ 18 years old and had ≥ 1 of 15 specific chronic diseases. Heat exposure was defined as thermal stress and operationalized by maximum temperatures and relative humidity assessed by 88 meteorological stations. Psychosocial factors were measured by standardized questionnaires assessing health literacy, self-efficacy, social support, risk expectations, and somatosensory amplification. Adverse effects of heat were operationalized by limitations in usual activities due to 14 specific symptoms reported at up to 12 follow-up assessments per participant. Data were analyzed by multivariable, multilevel, and mixed-effects linear regression.

**Results:**

A total of 4434 observations of 509 individuals were analyzed. Response rate was 28.1%. Participants had a mean age of 61.2 years (*SD* 13.7) and 240 participants (47.2%) identified themselves as women. Comparing the lowest range of heat exposure (11–15 °C) to the highest (37–40 °C), mean symptom burden increased by 79% from 3.7 (*SD* 5.3) to 6.7 (*SD* 6.0). Symptom burden was lower if participants reported better health literacy (− 0.15, 95% *CI* − 0.26/ − 0.05, *P* = 0.005), better general self-efficacy (− 0.20, 95% *CI* − 0.27/ − 0.14, *P* < 0.001), and perceived more social support (− 0.59, 95% *CI* − 1.07/ − 0.12, *P* = 0.015). Symptom burden was higher if participants reported more somatosensory amplification (0.18, 95% *CI* 0.13/0.24, *P* < 0.001) and expected a higher risk for adverse effects of heat (0.43, 95% *CI* 0.30/0.56, *P* < 0.001). We found significant effect modification (*P* = 0.041 through *P* < 0.001), indicating that the symptom burden related to light-to-moderate heat was more pronounced among patients with poorer psychosocial status.

**Conclusions:**

Light-to-moderate heat was associated with adverse effects. Health literacy, self-efficacy, and social support mitigated these effects, and negative expectations and the tendency to interpret benign bodily sensations as threatening amplified them.

**Trial registration:**

ClinicalTrials.gov NCT06407154.

**Supplementary Information:**

The online version contains supplementary material available at 10.1186/s12916-026-04622-4.

## Background

Despite the commitment of many nations to limit the global temperature increase, climate change is continuously progressing [1]. Global mean temperatures and the frequency, intensity, and duration of extreme weather events, such as heatwaves, are increasing worldwide [1, 2]. This phenomenon affects even temperate climate zones, such as Germany [3–5]. However, challenges for public health in these countries probably differ from those in hotter regions because of possible adaptive physiological responses to ambient temperatures [6].

Extreme heat is associated with higher mortality risks [7–9], but heat-related health complaints can also occur in mild or moderate forms, such as edema, muscle cramps, and nausea [10]. Therefore, public health already responds to light and moderate heat. For example, the risk categories of the US National Weather Service include “caution” (27 through 32 °C), “extreme caution” (32 through 40 °C), “danger” (40 through 51 °C), and “extreme danger” (more than 51 °C) [11].

In particular, people with chronic illnesses such as respiratory and cardiovascular diseases or long-standing diabetes mellitus are at risk of heat-related health problems, e.g., because the regulation of the core body temperature is physiologically impaired during hot weather [12–14]. The use of certain medications can also increase the risk of dehydration and heat-related illness [15]. Moreover, lower socioeconomic status and social isolation are important risk factors for heat-related mortality [16, 17]. As responsible authorities often fail to implement timely adaptation measures, health risks have to be addressed on an individual level [3].

Various stakeholders such as the World Health Organization and governmental and civil society organizations give recommendations on how vulnerable individuals should protect themselves from adverse effects of heat. For example, these recommendations include avoiding exposure to the sun and heat, avoiding physical exertion, and specific cooling strategies [18–20]. Clinical findings imply that such behavior could have protective effects [21], but its effect is probably limited and depends on the context [22–24]. Therefore, other strategies are also needed to mitigate adverse effects of heat. Psychosocial factors could be promising targets for intervention, because they are associated with the implementation and the effect of health-relevant behavior such as medication adherence [25].

Health literacy describes knowledge, motivation, and competences for accessing, understanding, appraising, and applying health information [26]. Self-efficacy relates to the ability or confidence to cope effectively with stressful events [27, 28]. Social support can be defined by social network, emotional support or acceptance, and supportive behavior in times of distress [29]. Somatosensory amplification describes the tendency to experience harmless bodily sensations as threatening [30, 31]. Elevated risk perceptions could be a psychological response to climate change [32]. Until now, few studies have analyzed the relationship between these factors and adverse effects of heat.

For this reason, our study aimed to observe adverse effects of light and moderate heat in individuals with chronic diseases and to analyze whether health literacy, self-efficacy, social support, risk expectations, and somatosensory amplification were associated with these effects. Light heat was defined by a heat exposure of > 27 through 32 °C and moderate heat by a heat exposure of > 32 through 40 °C. Adverse effects were operationalized by limitations in usual activities. Our hypothesis was that individuals with better health literacy, better self-efficacy, higher levels of perceived social support, lower risk expectations, and a lower level of somatosensory amplification would experience fewer adverse effects of heat.

## Methods

The CLIMATE study (“Chronical illness-related LIMitations in the Ability to cope with rising TEmperatures”) is a prospective cohort study based on an access-restricted online survey and publicly available weather data. Ethics approval was obtained from the Local Psychological Ethics Committee at the Center for Psychosocial Medicine of the University Medical Center Hamburg-Eppendorf (reference number LPEK-0605, approved on 1 May 2023 and amended on 1 May 2024). The study was piloted [33], prospectively registered on ClinicalTrials.gov (reference number NCT06407154), and reported using the Strengthening the Reporting of Observational Studies in Epidemiology (“STROBE”) checklist [34].

### Participant recruitment

Participants were recruited by 64 cooperating general practitioner (GP) practices. Patients of these practices were included if they were at least 18 years old, if they gave consent for study participation between 23 May and 15 September 2024, and if they had at least 1 out of 15 specific chronic diseases particularly susceptible to heat-related problems [7], including asthma, cardiac arrhythmias, chronic obstructive pulmonary disease, coronary heart disease, depressive disorder, type 1 diabetes mellitus, type 2 diabetes mellitus, heart failure, peripheral nervous system diseases, peripheral arterial occlusive disease, renal insufficiency, schizophrenia, state after myocardial infarction, state after transient ischemic attack, and state after stroke.

Patients were excluded if they had no capacity to consent, had severe visual impairment, had insufficient German language skills, or were not able to use an Internet browser. In each practice, up to 40 eligible patients received a leaflet containing a QR code and a short link facilitating registration for the online survey, as well as details on study aims, survey duration, collected data, location and duration of data storage, and investigator identity.

The survey was conducted using the web application Inquery (Inworks GmbH, Ulm, Germany). In the survey tool, the complete study details, including data protection measures, were shown before asking for informed consent. As an incentive, participants received a report summarizing their specific health complaints and their efforts against adverse effects of heat during each day of observation. This report was sent by email to the study participants after the survey was finished. On their own initiative, participants could share this report with their GP if they wanted to discuss how their protective behavior against heat could be improved. There was no data exchange between the study team and the recruiting practices.

### Data collection

After documenting informed consent within the application, patients received access to the baseline questionnaire. Subsequently, depending on the calendar date on which their baseline assessment was finalized, up to 12 invitations for follow-up were sent. Email invitations were connected with specific data sets. Therefore, no duplicate entries were generated. Invitations were sent out via email on the warmest days of each week at 6 pm. Specific days of observation were selected based on the maximum temperature that was expected in the respective week. Each unanswered invitation remained valid until the next invitation was sent. Therefore, participants could also provide their follow-up data on subsequent days.

Between 30 May and 25 September 2024, the weather forecast was checked every Thursday. If, in the upcoming 4 days, the maximum temperature was expected to exceed 30 °C, the warmest day in this time frame was chosen. Otherwise, the weather forecast was checked again on Monday to choose the warmest of the remaining days of the week. We excluded follow-up assessments during which participants were not in their hometown, because it was not possible to merge these survey data with specific weather data.

No log file analyses, time stamps, IP checks, or cookies were used. Incomplete questionnaires were not included in the final data set. The baseline questionnaire included 96 items, and the follow-up assessments included 31 items. Each web page within the survey tool contained only one item. For each item, answering was mandatory, but nonresponse options (e.g., “not applicable”) were provided and adaptive questioning (e.g., filter questions) was used.

The survey items were not randomized or altered between participants. Study participants could review and modify their answers before the questionnaire was finished. It was possible to leave the web pages and resume the questionnaires at a later time. Started questionnaires did not expire. Baseline data were collected between 23 May and 15 September 2024, and follow-up data were collected between 1 June and 19 September 2024.

### Dependent variable

Adverse effects of heat were operationalized by the severity of 14 specific symptoms reported at each follow-up including anxiousness, circulatory problems, confusion, depressiveness, dizziness, extrasystole, headache, muscle cramps, nausea, edemas, palpitations, shortness of breath, tiredness/fatigue, and vomiting. Severity was defined by the degree of limitations in usual activities during the last 24 h from 0 = “none” to 4 = “very severe, stopping (almost) all activities.” A summary score was calculated by adding the severity ratings of all specific symptoms [33]. The follow-up questionnaire can be found in Additional file 1.

### Heat exposure

Heat exposure was defined as thermal stress. On days with temperatures of 20 °C or more, heat exposure was operationalized by maximum heat index [35], which was calculated using hourly maximum temperature and hourly maximum relative humidity [36]. On days with temperatures below 20 °C, heat exposure was operationalized by maximum temperature, because heat index is not valid for these temperatures [37].

Maximum heat indices and maximum temperatures were calculated using hourly regional weather data downloaded from the website of Germany’s National Meteorological Service [38]. For each participant, we chose the active meteorological station with the smallest spatial distance to the center of the participant’s postal code area. Due to a possible lagged response to heat exposure, we used weather data of the preceding day if participants provided the follow-up before 2 pm. Otherwise, we used data of the day on which the follow-up was conducted.

For descriptive analyses, heat exposure was rounded to integer values and grouped into larger categories if they were represented by less than 40 observations, e.g., “37 through 40 °C.” Concordant with the risk levels used by the US National Weather Service, heat exposure was analyzed in the regression models in three categories, i.e., “no heat” (temperatures below 20 °C or heat index between 20 and 27 °C), “light heat” (heat index of more than 27 through 32 °C), and “moderate heat” (heat index of more than 32 through 40 °C) [11].

### Other independent variables

Sociodemographic data were reported at baseline and included age, sex, living arrangement, and educational level of participants as well as country of birth of participants and their parents. Educational level was operationalized in three categories using the CASMIN classification [39], i.e., “tertiary,” “secondary,” and “primary or below.”

Pursuant to a recent international consensus of 150 professionals and 25 public participants, multimorbidity was measured by a weighted disease count [40]. This measure was calculated using participant-reported severity of their chronic diseases at baseline by adding the defined severity points:

- Anxiety
  - No medication used (= 1)
  - Medication used (= 2)
- Asthma
  - Inhaler used only if necessary (= 1)
  - Inhaler used every day (= 2)
- Cardiac arrhythmias
  - Medication used (= 1)
  - Pacemaker used (= 2)
  - Medication and pacemaker used (= 3)
- Chronic obstructive pulmonary disease
  - Inhaler used (= 1)
  - Medication used (= 2)
- Coronary heart disease
  - No myocardial infarction in the past (= 1)
  - Myocardial infarction in the past (= 2)
- Depression
  - No medication used (= 1)
  - Medication used (= 2)
- Diabetes mellitus
  - Medication used (= 1)
  - Insulin used (= 2)
  - Insulin and medication used (= 3)
- Heart failure
  - Ordinary activity does not cause health complaints (= 1).
  - Ordinary activity causes health complaints (= 2).
  - Less-than-ordinary activity causes health complaints (= 3).
  - Health complaints due to heart failure even while at rest (= 4)
- Neuropathy
  - No regularly or persistently occurring symptoms (= 1)
  - Regularly or persistently occurring symptoms (= 2)
- Peripheral arterial occlusive disease
  - More than 200-m walking distance without pain (= 1)
  - Less than 200-m walking distance without pain (= 2)
- Renal insufficiency
  - No medication used and no dialysis necessary (= 1)
  - Medication used, but no dialysis necessary (= 2)
  - Dialysis necessary (= 3)
- Schizophrenia
  - No medication used (= 1)
  - Medication used (= 2)
- Transient ischemic attack and stroke
  - Transient ischemic attack, but no stroke (= 1)
  - Stroke with and without transient ischemic attack (= 2)

The questionnaires, which we used to assess sociodemographic data and multimorbidity, can be found in Additional file 2.

We also included several validated instruments at baseline representing psychosocial factors. Health literacy was measured by the HLS-EU-Q16 questionnaire, which contains 16 items with a 4-point Likert-scale. The number of items with positive rating is counted to calculate the summary score [41, 42]. Perceived social support was assessed using the F-SozU K14 questionnaire comprising 14 items, which are rated on a 5-point Likert-scale representing values from 1 to 5. The summary score is calculated by adding the individual values of each item and dividing by the number of answered items [43]. General self-efficacy is represented by a scale containing 10 items with a 4-point Likert scale indicating values from 1 through 4 [44, 45] and somatosensory amplification by a scale containing 10 items rated on a 5-point Likert scale indicating values from 0 through 4 [31, 46]. The summary scores of both scales are calculated by adding the individual points for each item. In addition, perceived risk for adverse effects of heat was reported using a numerical rating scale from 0 = “no risk” to 10 = “very high risk.” The respective questionnaire can be found in Additional file 2.

### Statistical analyses

Descriptive analyses were reported as numbers and percentages for categorical variables and means and standard deviations for continuous variables. We also reported pairwise Pearson correlations between independent variables. Relevant correlations were defined by *r* ≥ 0.3. The change in symptom burden (dependent variable) due to heat exposure (independent variable) was analyzed using univariable linear regression.

Associations between independent variables and the dependent variable were analyzed by unadjusted and multivariable multilevel linear regression assuming an independent covariance structure and adjusted for nested random effects on the meteorological station and the study participant within meteorological station levels. Respective effect measures were reported with corresponding 95% confidence intervals.

Dependent variable in all models was the summary score of symptom severity. Each unadjusted model contained one of the following independent variables: age (in years), sex (men vs. women), living arrangement (living together with others vs. living alone), educational level (tertiary vs. secondary, tertiary vs. primary or below), country of birth (participant and parents born in Germany vs. participant born in Germany and at least one parent born abroad, participant and parents born in Germany vs. participant born abroad), weighted disease count, health literacy summary score, general self-efficacy summary score, perceived social support summary score, somatosensory amplification summary score, and numerical rating of expected risk for adverse effects of heat. The multivariable model contained all of these independent variables. In addition, all unadjusted and multivariable models were controlled for heat exposure and the follow-up number.

Using the same models, we also analyzed if the association between heat exposure, psychosocial factors, and symptom burden differed between observations with median or lower spatial distance and observations with higher than median spatial distance between meteorological stations and participants’ homes. In addition, we also calculated our statistical models with a log-transformed dependent variable as sensitivity analyses.

Interaction terms between psychosocial factors and heat exposure were analyzed in a separate statistical model for each factor adjusted for age, sex, living arrangement, educational level, country of birth, and weighted disease count. For these analyses, interaction *p*-values, and stratified effect measures with 95% confidence intervals were reported. In addition, we presented the marginal adjusted means and mean differences at two defined values of each psychosocial factor (i.e., 25% percentile and 75% percentile, each).

All analyses used the available data set, i.e., we did not impute missing values or missing observations. Also, the natural sample was used without weighting or propensity scores. An alpha level of 5% (*P* < 0.05) was defined as statistically significant. Statistical analyses were performed using Stata 15.1.

## Results

### Recruitment

**Fig. 1:**
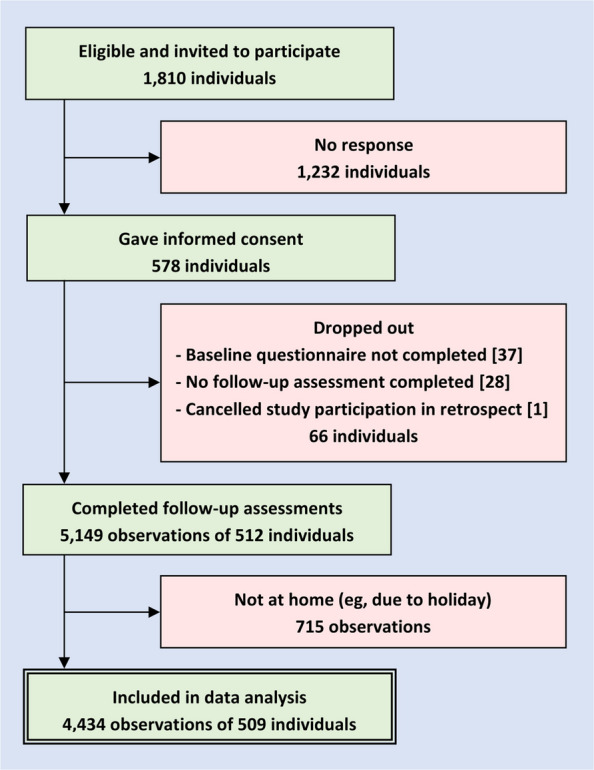
Recruitment of participants

Participant recruitment is shown in Fig. 1. Of 1810 contacted individuals, 509 (28.1%) gave informed consent, participated at baseline, and provided at least one follow-up included in data analysis. Following 5396 invitations, 5149 follow-up assessments (95.4%) were realized, and 4434 of them were made when participants were in their hometown and therefore included. On average, 8.7 (standard deviation, *SD* 3.3) observations per participant could be included with a median of 10 (interquartile range, *IQR* 7–12) observations. The maximum of 12 included observations was provided by 131 (25.7%) participants, while 16 (3.1%) provided only 1 (cf. Additional file 3: Fig. S1).

**Fig. 2:**
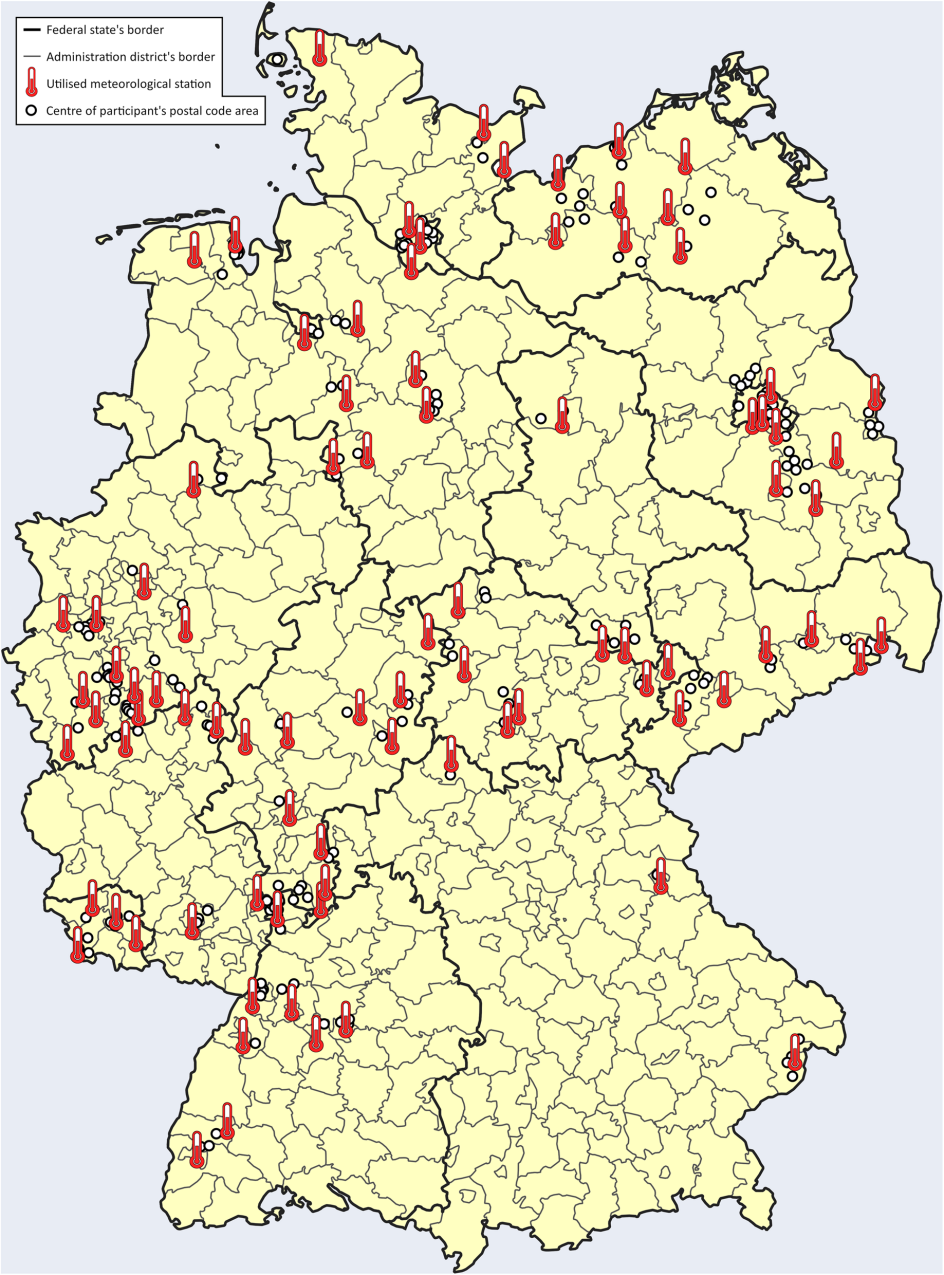
Spatial distribution of study participants and utilized meteorological stations across Germany’s federal states and administration districts. ©GeoBasis-DE/German Federal Agency for Cartography and Geodesy (BKG) 2024 (modified)

On average, each practice recruited 8.0 (*SD* 5.8) participants with a median of 6 (*IQR* 4–9.5). Participants lived in 79 of the 400 German administration districts, and all 16 German federal states were represented in the data (cf. Fig. 2). Using categories by the German Federal Institute for Research on Building, Urban Affairs and Spatial Development [47], 147 (28.9%) participants lived in sparsely populated rural districts, 78 (15.3%) in rural districts with signs of agglomeration, 156 (30.6%) in urban districts, and 128 (25.1%) in administratively independent cities. Mean spatial distance between the center of participants’ postal code area and the respective meteorological stations was 11.4 km (*SD* 6.7 km) with a median of 11 km (*IQR* 6–16 km).

### Baseline data

Sociodemographic data and chronic diseases are shown in Tables 1 and 2. The participants’ mean age was 61.2 years (*SD* 13.7 years, median 62 years, *IQR* 54–71 years), 240 participants (47.2%) identified themselves as women, and 115 (22.6%) were living alone. The educational level of 148 (29.1%) participants was tertiary, and 91 (17.9%) reported primary level or below. Nine out of 10 participants (461, 90.6%) were born in Germany and had German-born parents. The most prevalent chronic diseases were diabetes mellitus (180, 35.4%), depression (141, 27.7%), coronary heart disease (136, 26.7%), asthma (124, 24.4%), and heart failure (121, 23.8%). On average, participants had 2.3 (*SD* 1.6) and a median of 2 (*IQR* 1–3) of the 15 selected chronic diseases. The weighted disease count had a mean of 3.5 (*SD* 2.6) and a median of 3 (*IQR* 2–5).

**Table 1:** Sociodemographic data (*n* = 509)

Characteristic	Value
Age in years: mean	61.2 (SD 13.7)
Sex	
Men	269 (52.9%)
Women	240 (47.2%)
Living arrangement	
Living alone	115 (22.6%)
Living together with others, thereof (multiple entries possible):	394 (77.4%)
* Married or cohabiting*	*357 (90.6%)*
* Living together with own children or children of partner*	*88 (22.3%)*
* Living together with own parents or parents of partner*	*23 (5.8%)*
* Living together with other family members*	*16 (4.1%)*
* Living together with others, e.g., in a shared flat*	*11 (2.8%)*
Educational level	
Tertiary	148 (29.1%)
Secondary	270 (53.1%)
Primary or below	91 (17.9%)
Country of birth	
Participant and parents born in Germany	461 (90.6%)
Participant born in Germany, at least one parent abroad	27 (5.3%)
Participant born abroad	21 (4.1%)

Unless otherwise specified, data are reported as number (percent) of participants

*n* number of participants, *SD* standard deviation

**Table 2:** Chronic diseases (*n* = 509)

Characteristic	Value
Diabetes mellitus, thereof:	180 (35.4%)
Medication used	99 (19.4%)
Insulin used	43 (8.4%)
Insulin and medication used	38 (7.5%)
Depression, thereof:	141 (27.7%)
No medication used	62 (12.2%)
Medication used	79 (15.5%)
Coronary heart disease, thereof:	136 (26.7%)
No myocardial infarction in the past	64 (12.6%)
Myocardial infarction in the past	72 (14.2%)
Asthma, thereof:	124 (24.4%)
Inhaler used only if necessary	68 (13.4%)
Inhaler used every day	56 (11.0%)
Heart failure, thereof:	121 (23.8%)
Ordinary activity does not cause health complaints	51 (10.0%)
Ordinary activity causes health complaints	70 (13.8%)
Cardiac arrhythmias, thereof:	105 (20.6%)
Medication used	50 (9.8%)
Pacemaker used	15 (3.0%)
Medication and pacemaker used	40 (7.9%)
Anxiety, thereof:	87 (17.1%)
No medication used	53 (10.4%)
Medication used	34 (6.7%)
Neuropathy, thereof:	74 (14.5%)
Without regularly or persistently occurring symptoms	33 (6.5%)
With regularly or persistently occurring symptoms	41 (8.1%)
Chronic obstructive pulmonary disease, thereof:	63 (12.4%)
Inhaler used	4 (0.8%)
Medication used	59 (11.6%)
Peripheral arterial occlusive disease, thereof:	46 (9.0%)
More than 200-m walking distance without pain	34 (6.7%)
Less than 200-m walking distance without pain	12 (2.4%)
Transient ischemic attack and stroke, thereof:	46 (9.0%)
Transient ischemic attack, but no stroke	21 (4.1%)
Stroke (with and without transient ischemic attack)	25 (4.9%)
Renal insufficiency, thereof:	38 (7.5%)
No medication used and no dialysis necessary	27 (5.3%)
Medication used, but no dialysis necessary	11 (2.2%)
Schizophrenia, no medication used	1 (0.2%)

Data are reported as number (percent) of participants

*n* number of participants

The distributions of dependent variable and continuous independent variables are shown in Additional file 3: Fig. S2. Mean health literacy was 11.8 (*SD* 3.2, median 12, *IQR* 10–15) on a scale from 0 to 16, mean general self-efficacy 29.6 (*SD* 5.3, median 30, *IQR* 27–33) on a scale from 10 to 40, mean perceived social support 4.1 (*SD* 0.7, median 4.1, *IQR* 3.7–4.6) on a scale from 1 to 5, and mean somatosensory amplification 20.7 (*SD* 6.0, median 21, *IQR* 17–25) on a scale from 0 to 40. On a scale from 0 to 10, mean expected risk for adverse effects of heat was 3.7 (*SD* 2.6, median 3, *IQR* 2–6).

Pairwise correlations between independent variables can be found in Additional file 3: Table S1. There was an inverse correlation between living alone and perceived social support (*r* = − 0.31, *P* < 0.001). Perceived social support was also associated with health literacy (*r* = 0.36, *P* < 0.001), health literacy with general self-efficacy (*r* = 0.40, *P* < 0.001), and general self-efficacy with perceived social support (*r* = 0.37, *P* < 0.001). Additionally, somatosensory amplification was associated with expected risk for adverse effects of heat (*r* = 0.38, *P* < 0.001) and with identifying as woman (*r* = 0.30, *P* < 0.001).

### Follow-up data

Mean maximum daily heat exposure between 1 June and 19 September 2024 varied between the 88 utilized meteorological stations from 19.4 °C (*SD* 5.6 °C) at Schmücke to 26.2 °C (*SD* 4.6 °C) at Rheinstetten (cf. Additional file 3: Fig. S3). Most observations (3510, 79.2%) were made during maximum heat exposure between 25 and 33 °C (cf. Additional file 3: Fig. S4). Heat exposure of 27 °C or less was represented in 1446 (32.6%) observations, heat exposure of more than 27 through 32 °C in 2351 (53.0%) observations, and heat exposure of more than 32 through 40 °C in 637 (14.4%) observations.

**Fig. 3:**
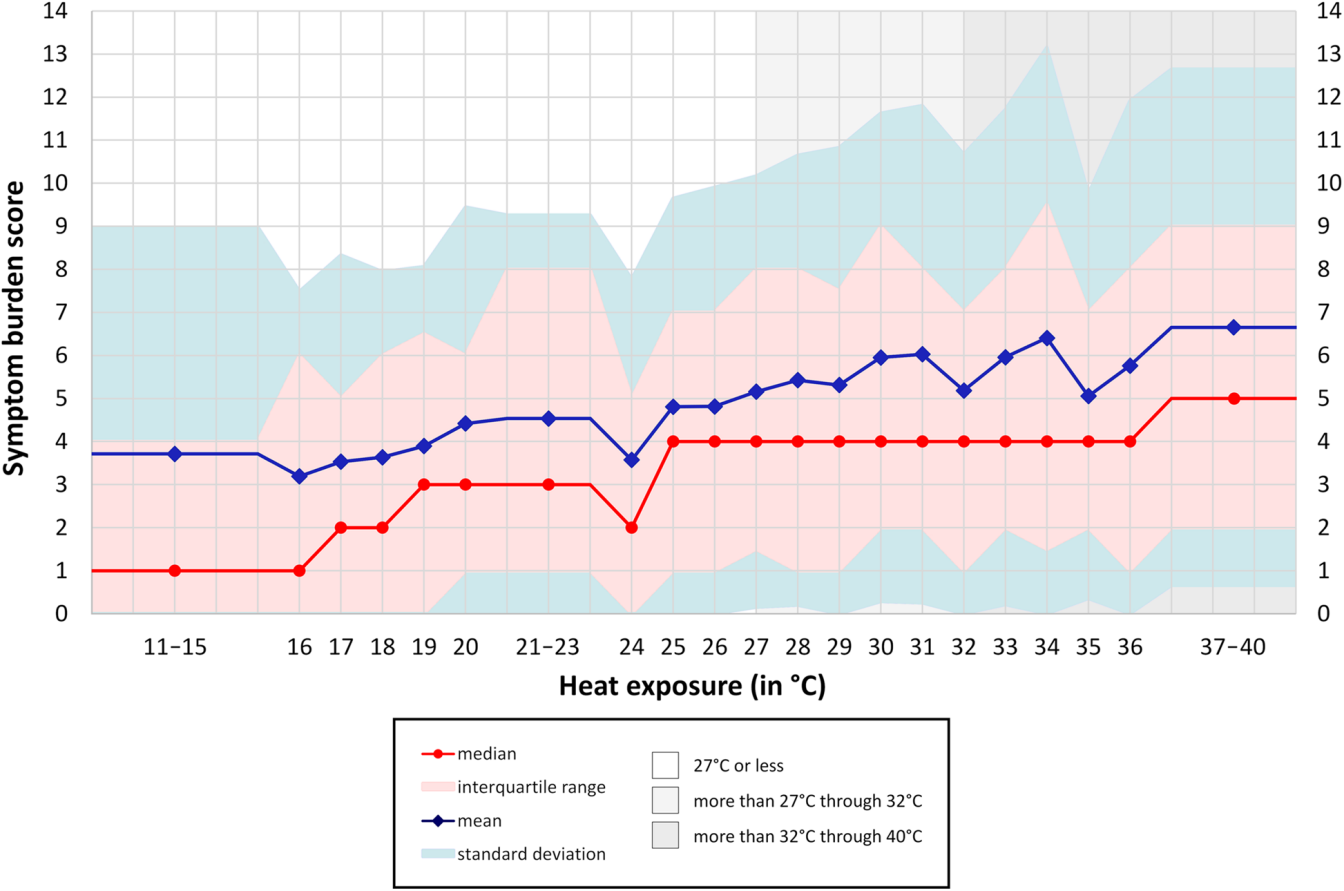
Symptom burden score by heat exposure (*N* = 4434). N, number of observations

Symptom burden had a mean of 5.2 (*SD* 5.4), a median of 4 (*IQR* 1 − 8), and a range from 0 to 38. The association between heat and symptom burden is shown in Fig. 3. On average, an increase in heat exposure by 10 °C was associated with an increase in symptom burden by 1 point (1.06, 95% confidence interval (95% CI), 0.81 − 1.31, *P* < 0.001). Comparing the lowest range of heat exposure (11 through 15 °C) to the highest (37 through 40 °C), mean symptom burden increased by 79% from 3.7 (*SD* 5.3) to 6.7 (*SD* 6.0), and the median of this variable increased from 1 (*IQR* 0 − 4) to 5 (*IQR* 2 − 9).

**Fig. 4:**
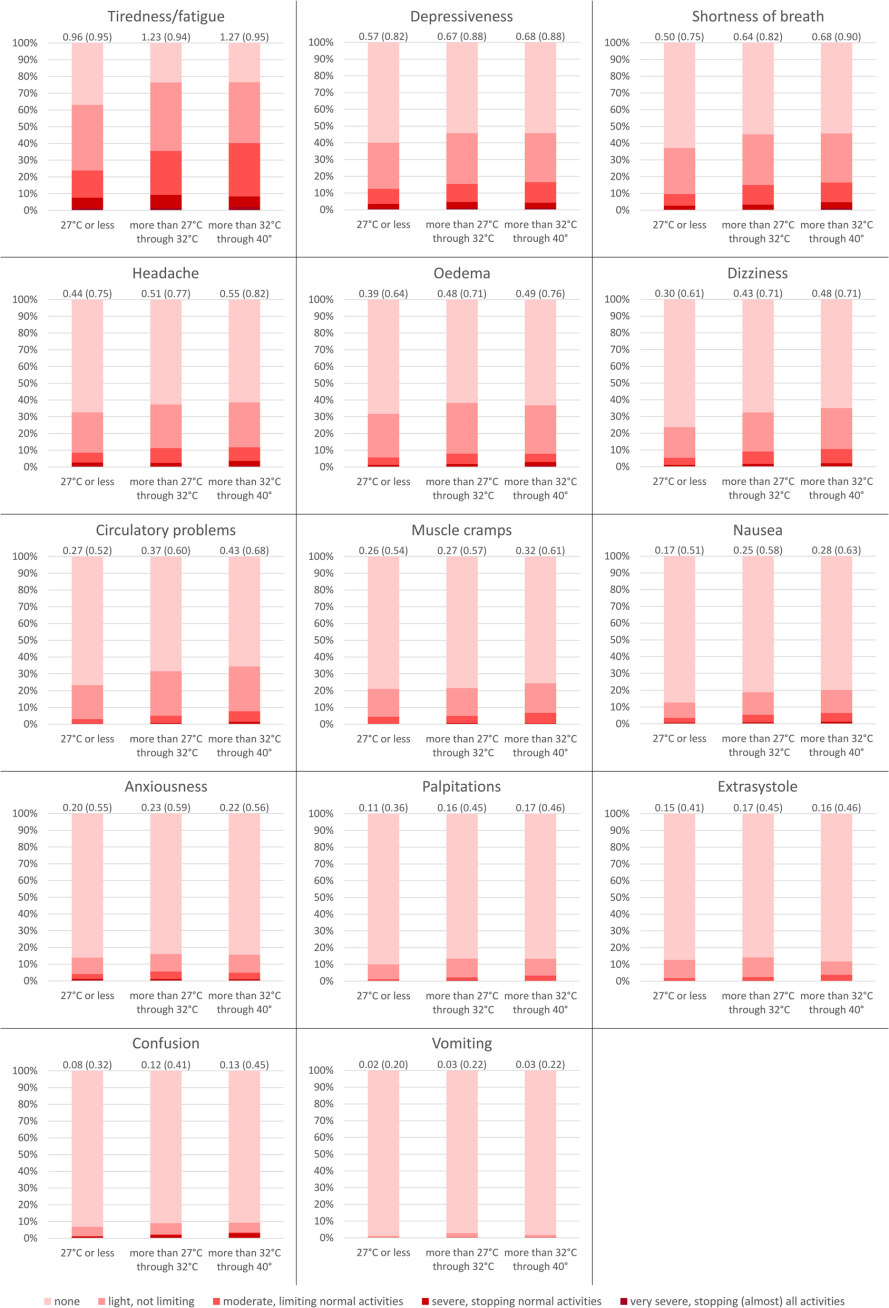
Severity of specific symptoms by heat exposure: mean (standard deviation) (*N* = 4434)

These increases were reflected on the level of the specific symptoms (cf. Fig. 4). For example, during a heat exposure of 27 °C or less, participants reported tiredness/fatigue in 912 of 1446 observations (61.3%) and during a heat exposure of > 32 through 40 °C in 488 of 637 observations (76.6%). The biggest increases in the mean symptom severity were observed for nausea (+ 46.2% at > 27 °C through 32 °C and + 63.2% at > 32 through 40 °C compared to 27 °C or less), confusion (+ 45.6%, + 63.0%), and circulatory problems/loss of consciousness (+ 39.2%, + 62.5%).

### Unadjusted models

Results from the unadjusted multilevel linear regression analyses are shown in Table 3. Compared to an exposure of 27 °C or less, a heat exposure of > 27 through 32 °C (1.06, 95% confidence interval (CI) 0.86/1.26, *P* < 0.001) was associated with a higher symptom burden. This association was even stronger at a heat exposure of > 32 through 40 °C (1.49, 95% *CI* 1.21/1.78, *P* < 0.001). Symptom burden was also higher in patients identifying as woman (1.36, 95% *CI* 0.57/2.15, *P* = 0.001) and reporting secondary (1.49, 95% *CI* 0.58/2.40, *P* = 0.001) or primary education or below (1.79, 95% *CI* 0.60/2.98, *P* = 0.003) instead of tertiary education.

**Table 3:** Association of symptom burden with heat and participant characteristics: results of linear regression analyses^a^ (*n* = 509, *N* = 4434)

	**Unadjusted models**^**b**^		**Multivariable model**	
**Characteristic**	**Coefficient (95% CI)**	***P***	**Coefficient (95% CI)**	***P***
Heat exposure				
27 °C or less	Reference		Reference	
More than 27 through 32 °C	1.06 (0.86/1.26)	< 0.001	1.07 (0.87/1.27)	< 0.001
More than 32 through 40 °C	1.49 (1.21/1.78)	< 0.001	1.51 (1.22/1.79)	< 0.001
Age	− 0.03 (− 0.06/0.003)	0.075	− 0.004 (− 0.03/0.02)	0.717
Sex				
Men	Reference		Reference	
Women	1.36 (0.57/2.15)	0.001	0.30 (− 0.36/0.95)	0.370
Living arrangement				
Living together with others	Reference		Reference	
Living alone	0.84 (− 0.11/1.79)	0.083	− 0.18 (− 0.95/0.58)	0.636
Educational level				
Tertiary	Reference		Reference	
Secondary	1.49 (0.58/2.40)	0.001	0.61 (− 0.10/1.31)	0.090
Primary or below	1.79 (0.60/2.98)	0.003	0.59 (− 0.33/1.51)	0.210
Country of birth				
Participant and parents born in Germany	Reference		Reference	
Participant born in Germany, at least one parent abroad	− 0.75 (− 2.52/1.03)	0.409	− 0.54 (− 1.86/0.78)	0.424
Participant born abroad	− 1.51 (− 3.55/0.53)	0.146	− 1.95 (− 3.48/− 0.41)	0.013
Weighted disease count	0.59 (0.44/0.73)	< 0.001	0.31 (0.19/0.44)	< 0.001
Health literacy	− 0.50 (− 0.62/− 0.39)	< 0.001	− 0.15 (− 0.26/− 0.05)	0.005
General self-efficacy	− 0.39 (− 0.46/− 0.32)	< 0.001	− 0.20 (− 0.27/− 0.14)	< 0.001
Perceived social support	− 1.95 (− 2.47/− 1.44)	< 0.001	− 0.59 (− 1.07/− 0.12)	0.015
Somatosensory amplification	0.35 (0.29/0.41)	< 0.001	0.18 (0.13/0.24)	< 0.001
Expected risk for adverse effects of heat	0.81 (0.68/0.95)	< 0.001	0.43 (0.30/0.56)	< 0.001

^a^Controlled for observation numbers and adjusted for random effects on the levels of meteorological stations and participants

^b^Also controlled for heat exposure

*CI* confidence interval, *n* number of participants, *N* number of observations

Moreover, in the unadjusted analyses, a higher number and severity of chronic diseases indicated by a higher weighted disease count (0.59, 95% *CI* 0.44/0.73, *P* < 0.001), a higher degree of somatosensory amplification (0.35, 95% *CI* 0.29/0.41, *P* < 0.001), and a higher expected risk for adverse effects of heat (0.81, 95% *CI* 0.68/0.95, *P* < 0.001) were also associated with a higher symptom burden. Conversely, better health literacy (− 0.50, 95% *CI* − 0.62/− 0.39, *P* < 0.001), better general self-efficacy (− 0.39, 95% *CI* − 0.46/− 0.32, *P* < 0.001), and perceptions of better social support (− 1.95, 95% *CI* − 2.47/− 1.44, *P* < 0.001) were associated with lower symptom burden.

### Multivariable model

Results from the multivariable multilevel linear regression analysis can also be found in Table 3. The effect of heat exposure on symptom burden only mildly changed in the multivariable model (> 27 through 32 °C: 1.07, 95% *CI* 0.87/1.27, *P* < 0.001, and > 32 through 40 °C: 1.51, 95% *CI* 1.22/1.79, *P* < 0.001). However, female sex (*P* = 0.370) and the educational level (*P* = 0.090 and *P* = 0.210, respectively) lost their association with symptom burden. In contrast to the unadjusted analyses, we found an association between being born abroad (− 1.95, 95% *CI* − 3.48/− 0.41, *P* = 0.013) and a lower symptom burden in the multivariable model. Moreover, the effect of the weighted disease count decreased sharply (0.31, 95% *CI* 0.19/0.44, *P* < 0.001).

In the multivariable model, the effects of all psychosocial scales were also considerably reduced. However, adjusted for the other variables, symptom burden remained lower if participants reported better health literacy (− 0.15, 95% *CI* − 0.26/− 0.05, *P* = 0.005), better general self-efficacy (− 0.20, 95% *CI* − 0.27/− 0.14, *P* < 0.001), and perceived a higher level of social support (− 0.59, 95% *CI* − 1.07/− 0.12, *P* = 0.015). Symptom burden still was higher if participants reported more somatosensory amplification (0.18, 95% *CI* 0.13/0.24, *P* < 0.001) and expected a higher risk for adverse effects of heat (0.43, 95% *CI* 0.30/0.56, *P* < 0.001).

### Analyses of interactions

**Fig. 5:**
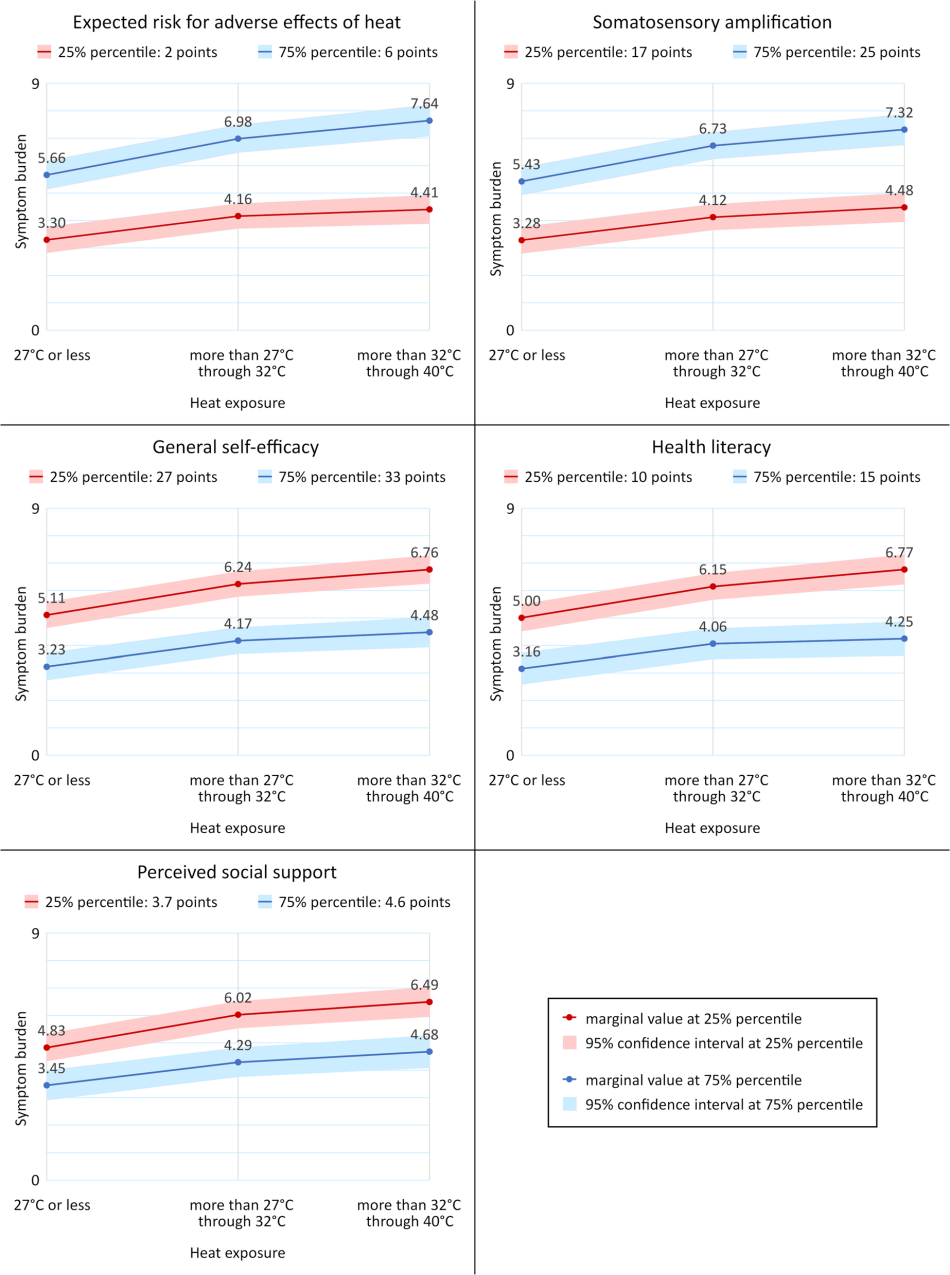
Average adjusted means in symptom burden in heat exposure strata at defined levels of psychosocial factors: results of linear regression analyses^a^ (*n* = 509, *N* = 4434). ^a^Controlled for observation numbers, age, sex, living arrangement, educational level, country of birth, and weighted disease count and adjusted for random effects on the levels of meteorological stations and participants. n, number of participants; N, number of observations

The effect of heat exposure on symptom burden is modified by all psychosocial factors (*P* = 0.041 through *P* < 0.001). Compared to 27 °C and less, heat exposure of more than 27 through 32 °C has a slightly larger effect, and heat exposure of more than 32 to 40 °C has a much larger effect in patients whose health literacy, self-efficacy, or social support scores are at the 25% percentile than in patients with scores at the 75% percentile. The same effect modification was found for patients whose somatosensory amplification or risk expectation scores are at the 75% percentile compared to patients with these scores at the 25% percentile. The respective mean differences in symptom burden in heat exposure strata are shown at the defined levels of psychosocial factors in Table 4, and the respective average adjusted means are demonstrated at these levels in Fig. 5.

**Table 4:** Association between heat exposure and symptom burden at defined levels of psychosocial factors: results of linear regression analyses^a^ (*n* = 509, *N* = 4434)

Characteristic	Coefficient (95% CI)	*P*_A_	Coefficient (95% CI)	*P*_A_	*P*_I_
	**Health literacy** **at 25% percentile (10 points)**		**Health literacy** **at 75% percentile (15 points)**		
Heat exposure 27 °C or less More than 27 through 32 °C More than 32 through 40 °C	Reference 1.14 (0.91/1.37) 1.76 (1.43/2.10)	< 0.001 < 0.001	Reference 0.91 (0.63/1.19) 1.09 (0.70/1.48)	< 0.001 < 0.001	0.012
	**General self-efficacy** **at 25% percentile (27 points)**		**General self-efficacy** **at 75% percentile (33 points)**		
Heat exposure 27 °C or less More than 27 through 32 °C More than 32 through 40 °C	Reference 1.13 (0.91/1.35) 1.66 (1.34/1.97)	< 0.001 < 0.001	Reference 0.94 (0.71/1.18) 1.25 (0.91/1.59)	< 0.001 < 0.001	0.041
	**Perceived social support** **at 25% percentile (3.7 points)**		**Perceived social support** **at 75% percentile (4.6 points)**		
Heat exposure 27 °C or less More than 27 through 32 °C More than 32 through 40 °C	Reference 1.20 (0.97/1.42) 1.67 (1.35/1.98)	< 0.001 < 0.001	Reference 0.84 (0.59/1.09) 1.23 (0.87/1.58)	< 0.001 < 0.001	0.007
	**Somatosensory amplification** **at 25% percentile (17 points)**		**Somatosensory amplification** **at 75% percentile (25 points)**		
Heat exposure 27 °C or less More than 27 through 32 °C More than 32 through 40 °C	Reference 0.84 (0.60/1.08) 1.20 (0.87/1.52)	< 0.001 < 0.001	Reference 1.30 (1.06/1.55) 1.89 (1.53/2.25)	< 0.001 < 0.001	< 0.001
	**Expected risk for** **adverse effects of heat** **at 25% percentile (2 points)**		**Expected risk for** **adverse effects of heat** **at 75% percentile (6 points)**		
Heat exposure 27 °C or less More than 27 through 32 °C More than 32 through 40 °C	Reference 0.87 (0.63/1.10) 1.11 (0.77/1.45)	< 0.001 < 0.001	Reference 1.32 (1.06/1.59) 1.98 (1.61/2.35)	< 0.001 < 0.001	< 0.001

^a^Controlled for observation numbers,age, sex, living arrangement, educational level, country of birth, and weighted disease count and adjusted for random effects on the levels of meteorological stations and participants

*CI* confidence interval, *n* number of participants, *N* number of observations, *P*_A_ *P*-value of association with symptom burden, *P*_I_ *P*-value of interaction with heat index

### Sensitivity analyses

In the unadjusted models for both subgroups defined by spatial distance, we identified associations with the same variables as in the main analyses. However, the effect sizes of heat exposure, sex, educational level, health literacy, somatosensory amplification, and the expected risk for adverse effects of heat were slightly larger in observations with higher distance. In addition, living arrangement was associated with symptom burden in observations with higher distance, but not in the main analyses or in observations with lower distance (cf. Additional file 3: Table S2).

In the multivariable models, there also was a high level of concordance between main analyses and both analyses of subgroups defined by spatial distance. However, compared to the unadjusted models, health literacy and perceived social support lost their associations with symptom burden in observations with smaller distance, which did not happen in the main analyses or in observations with higher distance. In addition, the effect sizes of heat exposure and expected risk for adverse effects of heat were slightly larger in observations with higher distance, country of birth lost its association, and an association of the educational level with symptom burden could be identified that was not found in observations with smaller distance or in the main analyses (cf. Additional file 3: Table S3).

In unadjusted and multivariable models with log-transformed dependent variable, the same variables were associated with symptom burden as in the main analyses. However, we identified an additional association with age in the unadjusted log-transformed analyses, which was not found in the main analyses (cf. Additional file 3: Table S4).

## Discussion

Under everyday conditions, individuals with chronic diseases were vulnerable to adverse effects of light and moderate heat exposure. Independently of heat exposure, lower levels of health literacy, self-efficacy and social support, more negative expectations regarding the effects of heat, and a higher degree of somatosensory amplification were associated with a higher symptom burden. Furthermore, we found significant effect modification, indicating that the symptom burden related to light and moderate heat was more pronounced among patients with poorer psychosocial status.

### Comparison with the literature

Consistent with our results, other studies also reported more health complaints on days with higher temperatures and a higher symptom burden in individuals with a higher number and more severe chronic diseases [12, 14, 48]. Social support and social participation were associated with lower risk of heat-related mortality, emergency department visits, and hospitalizations [48, 49]. Better heat-related health literacy was associated with fewer unscheduled hospital visits [50]. In other contexts than heat, studies also reported an association between a better self-efficacy and lower severity of symptoms [51–54]. Interpreting physical symptoms as controllable and manageable can lead to perceptions of lower symptom severity and a higher likeliness for adaptive coping behaviors [51, 55].

In addition to adverse physical effects of heat, perceived symptom burden is also influenced by cognitive and emotional processing of heat and its effects. Generally, the way in which symptoms are perceived and interpreted is associated with self-reported severity of health problems. For example, physical symptoms are intensified if they are attributed to a serious illness and not to more harmless causes [56]. Thus, somatosensory amplification is associated with a higher number of symptoms like pain and fatigue [33, 57–60]. The progression of climate change, rising temperatures, and their effects on health are worrying many individuals [61]. Increased attention and anxiety can also exacerbate symptoms as it can lead to symptoms being perceived as more threatening and dangerous [56].

### Implications

Interventions to strengthen self-efficacy and social support, and to limit risk perceptions and symptom amplification, could be an important approach to reduce the negative health consequences of heat in people with chronic diseases. For example, tailored nurse-led interventions facilitated the improvement of self-efficacy in patients with type 2 diabetes mellitus, metabolic syndrome, and with chronic back pain [62–64]. Moreover, digital health technologies could be used to elevate self-efficacy [65, 66], e.g., by using automatic heat warnings [67].

Interventions using cognitive behavioral therapy (CBT) also showed an improvement in self-efficacy, e.g., in patients with chronic low back pain, cardiovascular disease, and with fibromyalgia [68–70]. On an individual level, CBT could also potentially be a helpful intervention to reduce amplification tendencies in the perception of heat-related symptoms. For example, CBT has been effective in patients with chronic back pain or with fibromyalgia [71, 72].

Furthermore, interventions can improve health literacy in order to increase the knowledge and ability of individuals with chronic illnesses to protect themselves from the negative effects of heat. For example, in a systematic review, 15 of the 22 identified interventions were effective in improving functional, interactive, and critical health literacy, respectively [26]. There are also promising results suggesting that interventions such as a heat education program led by community health worker could improve heat literacy and reduce unscheduled hospital visits [50].

### Strengths and limitations

We conducted an observational study which is not suitable for confirming or rejecting effects of the analyzed factors but rather supports or questions existing hypotheses. Independent variables such as perceived risk for adverse effects of heat, which mediated the association between heat and symptom burden, were only measured at baseline. Thus, changes in these variables during the observation time could not be considered. Moreover, adverse effects of heat were defined by limitations in usual activities. Other concepts of symptom burden such as psychological suffering or worrying about death and disease [73] are not represented in our data.

It needs to be noted that data such as use of air-conditioning in the patients’ homes were not collected in our study. Therefore, we could not analyze which variables explained the associations between psychosocial factors on adverse effects of heat. Also, the set of control variables was limited, and possible confounding by variables such as cooling access and different medication profiles could not be considered. Our study identified many strong associations in the data set, but there was no sample size calculation. Therefore, it is possible that statistical power was not sufficient to detect all relevant associations. The awareness of being observed could have led to social desirable answers and affected the results. For example, underreporting of psychosocial barriers could have reduced the strength of associations that we found in our study [74].

The low response rate of 28% could point to a selection bias. For example, healthier and more health-literate individuals may have been more likely to participate. We also found that representativeness of our sample for the general population was limited in terms of sociodemographic data. In particular, there was a higher rate of participants with tertiary education (29% in our sample vs. 19% in the general population [75]) and a lower rate of participants with migration background (9% vs. 30% [76]). These differences might be related to our study design. For example, the use of an online survey could have increased the fraction of individuals with tertiary education. And using German language for patient education and survey could have lowered the proportion of individuals with migration background.

Due to a limited number of days with heat exposure of more than 32 °C during summer in Germany [77], days of observation were not randomly selected. Instead, we invited participants to provide their follow-ups on the warmest day of each week. On the one hand, this ensured that more than 14% of the observations were made during heat exposure of 32 °C or more. On the other hand, the number of observations on cold days therefore are underrepresented in our data. While 33% of the observations were made during 27 °C or less, which constitutes our reference category, only 1% of the observations were made during 15 °C or less. Therefore, our study does not inform about the effect of colder ambient temperatures on health complaints. It was also not possible to explore the effect of temporal variations, e.g., if repeated heat worsens symptoms over time, or if psychosocial factors have stronger effects early vs. late in heat season.

We used weather data from meteorological stations, which is the most accurate available data source. On average, there was a spatial distance of 11 km between meteorological stations and the participants’ homes. This rather large distance could have biased the heat exposure represented in our models. Sensitivity analyses identified a high level of concordance but also slight differences between observations with higher distance and observations with lower distance between meteorological stations and participants’ homes. Therefore, effect sizes of associations between heat exposure, psychosocial factors, and symptom burden should be interpreted with care.

Strengths of our study were inclusion of rural and urban areas and representation of all German federal states. Recruitment of participants was guided by defined inclusion and exclusion criteria, which is a better way to recruit a specific target population than self-selection of participants [78, 79]. Health complaints were assessed on the day of heat exposure; therefore, recall bias is unlikely. All survey data were measured with validated and established questionnaires. The data set had no missing values. There also was little loss to follow-up as 95% of the intended follow-up assessments could be realized. We used robust statistical analyses adjusting for confounding and clustering in the data set. Furthermore, the study was piloted and prospectively registered, which increased the robustness of the study design.

## Conclusions

We observed an association between heat and an increase in symptom burden. In accordance with our pre-specified hypotheses, adverse effects of heat were mitigated by better health literacy, better self-efficacy, and higher levels of social support, and they were intensified by negative risk expectations and tendencies to amplify benign bodily sensations. Individuals who have a higher risk for adverse effects of heat might benefit from interventions to address these factors (e.g., by tailored nurse-led interventions or health literacy education).

## Supplementary Information

Additional file 1. Follow-up questionnaire. Adverse effects of heat.

Additional file 2. Baseline questionnaire. (Multi-)Morbidity. Perceived risk for adverse effects of heat. Sociodemographic data.

Additional file 3: Supplementary figures and tables. Figure S1: Number of participants by number of observations. Figure S2: Distribution of dependent variable and continuous independent variables. Figure S3: Mean maximum daily heat exposure in °C between 1 July and 19 September 2024 by weather measuring station. Figure S4: Number of observations by heat exposure. Table S1: Pairwise correlations between independent variables. Table S2: Unadjusted association of symptom burden with heat and participant characteristics by distance between meteorological station and study participants’ homes. Table S3: Multivariable association of symptom burden with heat and participant characteristics by distance between meteorological station and study participants’ homes. Table S4: Association of log-transformed symptom burden with heat and participant characteristics.

Additional file 4.

## Data Availability

The datasets analysed during the current study are available from the corresponding author on reasonable request.

## Abbreviations

CBT: Cognitive behavioral therapy
CI: Confidence interval
CLIMATE: Chronical illness-related LIMitations in the Ability to cope with rising TEmperatures
GP: General practitioner
IQR: Interquartile range
SD: Standard deviation
STROBE: Strengthening the Reporting of Observational Studies in Epidemiology
UKE: Universitätsklinikum Hamburg-Eppendorf
