# Supplementary material for: Association between psychosocial factors and adverse effects of light-to-moderate ambient heat in patients with chronic diseases: results of the prospective cohort study CLIMATE-II
Source: BMC Med. 2026 Jan 15;24:52. doi: 10.1186/s12916-026-04622-4 (PMC12849320; doi:10.1186/s12916-026-04622-4)
Supplement: Supplementary file 1 — Additional file 1. Follow-up questionnaire. Adverse effects of heat. [file 12916_2026_4622_MOESM1_ESM.pdf]

ASSOCIATION BETWEEN PSYCHOSOCIAL FACTORS AND ADVERSE EFFECTS OF LIGHT-TO-MODERATE AMBIENT HEAT IN PATIENTS WITH CHRONIC DISEASES: RESULTS OF THE PROSPECTIVE COHORT STUDY CLIMATE-II.

Additional file 1: Follow-up questionnaire

Ingmar Schäfer, Valentina Paucke, Julia Nothacker, Agata Menzel, Susanne Döpfmer, Klaus Hager, Susann Hueber, Arian Karimzadeh, Thomas Kötter, Christin Löffler, Beate S. Müller, Martin Scherer, Dagmar Lühmann

Adverse effects of heat

Next, we would like to know whether you have experienced any of the following symptoms in the last 24 hours and how much they have affected your activities of daily living.

ST01 ... nausea

- ☐ no nausea.
- ☐ light nausea, not limiting.
- ☐ moderate nausea, limiting normal activities.
- ☐ severe nausea, stopping normal activities.
- ☐ very severe nausea, stopping (almost) all activities.

ST02 ... vomiting

- ☐ no vomiting.
- ☐ light form of vomiting, not limiting.
- ☐ moderate form of vomiting, limiting normal activities.
- ☐ severe form of vomiting, stopping normal activities.
- ☐ very severe form of vomiting, stopping (almost) all activities.

ST03 ... tiredness/fatigue

- ☐ no tiredness/fatigue.
- ☐ light tiredness/fatigue, not limiting.
- ☐ moderate tiredness/fatigue, limiting normal activities.
- ☐ severe tiredness/fatigue, stopping normal activities.
- ☐ very severe tiredness/fatigue, stopping (almost) all activities.

ST04 ... dizziness

- ☐ no dizziness.
- ☐ light dizziness, not limiting.
- ☐ moderate dizziness, limiting normal activities.
- ☐ severe dizziness, stopping normal activities.
- ☐ very severe dizziness, stopping (almost) all activities.

ST05 **… circulatory problems or loss of consciousness**

- ☐ no circulatory problems
- ☐ light circulatory problems without loss of consciousness , not limiting
- ☐ moderate circulatory problems with or without loss of consciousness, limiting normal activities.
- ☐ severe circulatory problems with or without loss of consciousness, stopping normal activities
- ☐ very severe circulatory problems with loss of consciousness, stopping (almost) all activities

ST06 **… muscle cramps**

- ☐ no muscle cramps.
- ☐ light muscle cramps, not limiting.
- ☐ moderate muscle cramps, limiting normal aktivities.
- ☐ severe muscle cramps, stopping normal activities.
- ☐ very severe muscle cramps, stopping (almost) all activities.

ST07 **… headache**

- ☐ no headache.
- ☐ light headache, not limiting.
- ☐ moderate headache, limiting normal aktivities.
- ☐ severe headache, stopping normal activities.
- ☐ very severe headache, stopping (almost) all activities.

ST08 **… extrasystole**

- ☐ no extrasystole.
- ☐ light extrasystole, not limiting.
- ☐ moderate extrasystole, limiting normal aktivities.
- ☐ severe extrasystole, stopping normal activities.
- ☐ very severe extrasystole, stopping (almost) all activities.

ST09	**… palpitations**

- ☐ no palpitations.
- ☐ light palpitations, not limiting.
- ☐ moderate palpitations, limiting normal aktivities.
- ☐ severe palpitations, stopping normal activities.
- ☐ very severe palpitations, stopping (almost) all activities.

ST10	**… edemas (e.g., in the legs)**

- ☐ no edemas.
- ☐ light edemas, not limiting.
- ☐ moderate edemas, limiting normal aktivities.
- ☐ severe edemas, stopping normal activities.
- ☐ very severe edemas, stopping (almost) all activities.

ST11	**… shortness of breath**

- ☐ no shortness of breath.
- ☐ light shortness of breath, not limiting.
- ☐ moderate shortness of breath, limiting normal aktivities.
- ☐ severe shortness of breath, stopping normal activities.
- ☐ very severe shortness of breath, stopping (almost) all activities.

ST12	**… depressiveness**

- ☐ no depressiveness.
- ☐ light depressiveness, not limiting.
- ☐ moderate depressiveness, limiting normal aktivities.
- ☐ severe depressiveness, stopping normal activities.
- ☐ very severe depressiveness, stopping (almost) all activities.

ST13 **… anxiousness**

- ☐ no anxiousness
- ☐ light anxiousness, not limiting.
- ☐ moderate anxiousness, limiting normal aktivities.
- ☐ severe anxiousness, stopping normal activities.
- ☐ very severe, stopping (almost) all activities.

ST14 **… confusion**

- ☐ no confusion.
- ☐ light confusion, not limiting.
- ☐ moderate confusion, limiting normal aktivities.
- ☐ severe confusion, stopping normal activities.
- ☐ very severe confusion, stopping (almost) all activities.
