# Supplementary material for: Association between psychosocial factors and adverse effects of light-to-moderate ambient heat in patients with chronic diseases: results of the prospective cohort study CLIMATE-II
Source: BMC Med. 2026 Jan 15;24:52. doi: 10.1186/s12916-026-04622-4 (PMC12849320; doi:10.1186/s12916-026-04622-4)
Supplement: Supplementary file 2 — Additional file 2. Baseline questionnaire. (Multi-)Morbidity. Perceived risk for adverse effects of heat. Sociodemographic data. [file 12916_2026_4622_MOESM2_ESM.pdf]

ASSOCIATION BETWEEN PSYCHOSOCIAL FACTORS AND ADVERSE EFFECTS OF LIGHT-TO-MODERATE AMBIENT HEAT IN PATIENTS WITH CHRONIC DISEASES: RESULTS OF THE PROSPECTIVE COHORT STUDY CLIMATE-II.

Additional file 2: Baseline questionnaire

Ingmar Schäfer, Valentina Paucke, Julia Nothacker, Agata Menzel, Susanne Döpfmer, Klaus Hager, Susann Hueber, Arian Karimzadeh, Thomas Kötter, Christin Löffler, Beate S. Müller, Martin Scherer, Dagmar Lühmann

(Multi-)Morbidity

CK01 Have you ever had a heart attack or been diagnosed with coronary heart disease?

- ☐ No.
- ☐ Yes, I have coronary heart disease, but I have not had a heart attack.
- ☐ Yes, I have had a heart attack.

CK02 Have you been diagnosed with heart failure?

- ☐ No.
- ☐ Yes, but I have (almost) no related symptoms.
- ☐ Yes, and I experience symptoms such as shortness of breath and chest tightness during strenuous physical activity, e.g. when climbing two flights of stairs.
- ☐ Yes, and I experience symptoms such as shortness of breath and chest tightness even during light physical activity, e.g. when walking on level ground.
- ☐ Yes, and I experience symptoms such as shortness of breath and chest tightness even when resting.

CK03 Have you been diagnosed with cardiac arrhythmia?

- ☐ No.
- ☐ Yes, but I do not take any medication for it and do not have a pacemaker.
- ☐ Yes, and I take medication for it, but I do not have a pacemaker.
- ☐ Yes, and I have a pacemaker.

CK04 Have you been diagnosed with intermittent claudication (arteriosclerosis in the legs) or PAOD (peripheral arterial occlusive disease)?

- ☐ No.
- ☐ Yes, but I only need to take a break when I walk 200 meters or more.
- ☐ Yes, and I need to take a break when I walk less than 200 meters.

CK05 Have you ever had a stroke or TIA (transient ischemic attack)?

- ☐ No.
- ☐ Yes I had a TIA, but I did not have a stroke.
- ☐ Yes, I had a stroke.

CK06 Have you been diagnosed with diabetes?

- [ ] No.
- [ ] Yes, but I don't need to take medication or insulin.
- [ ] Yes, and I need to take medication, but not insulin.
- [ ] Yes, and I need to take insulin.

CK07 Have you been diagnosed with chronic obstructive pulmonary disease (COPD)?

- [ ] No.
- [ ] Yes, and I use an inhaler for this, but I don't take any tablets.
- [ ] Yes, and I take tablets for this.

CK08 Have you been diagnosed with asthma?

- [ ] No.
- [ ] Yes, and I use an inhaler when needed.
- [ ] Yes, and I therefore use an inhaler every day.

CK09 Have you been diagnosed with kidney failure?

- [ ] No.
- [ ] Yes, but I don't need to take medication or have dialysis.
- [ ] Yes, and I therefore need to take medication, but I don't need dialysis.
- [ ] Yes, and I therefore need dialysis.

CK10 Have you been diagnosed with depression?

- [ ] No.
- [ ] Yes, but I don't have to take medication for it.
- [ ] Yes, and I have to take medication for it.

CK11 Have you been diagnosed with an anxiety disorder?

- [ ] No.
- [ ] Yes, but I don't have to take medication for it.
- [ ] Yes, and I have to take medication for it.

CK12	**Have you been diagnosed with schizophrenia?**

☐ No.

☐ Yes, but I don't have to take medication for it.

☐ Yes, and I have to take medication for it.

CK13	**Have you been diagnosed with neuropathies?**

☐ No.

☐ Yes, but I do not have regular or persistent symptoms as a result.

☐ Yes, and I have regular or persistent symptoms as a result.

Perceived risk for adverse effects of heat

Next, we would like to know how much you think heat can affect your health and how well you think it is possible to protect yourself against it.

IPR1	**How high do you estimate your personal risk of suffering health problems due to heat (e.g., shortness of breath, vomiting, loss of consciousness)?**

No risk	0	1	2	3	4	5	6	7	8	9	10	Very high risk
	☐	☐	☐	☐	☐	☐	☐	☐	☐	☐	☐	

Sociodemographic data

Finally, we would like to ask you for some personal details

SDD01 What is your gender?

- ☐ Female
- ☐ Male
- ☐ Non-binary

SDD02 In which year were you born?

⎿ ⏌⎿ ⏌⎿ ⏌⎿ ⏌

SDD04 Do you live alone?

- ☐ No
- ☐ Yes *(continue with SDD6)*

SDD05 Who else do you live with?
(Multiple answers possible)

- ☐ With my spouse or partner
- ☐ With my child or children
- ☐ With my own parents or my partner's parents
- ☐ With other family members
- ☐ With other people (e.g., acquaintances, roommates, etc.)

SDD08 What is your highest general school qualification?

- ☐ No school qualification
- ☐ Qualification from „Hauptschule“, „Volksschule“ oder or 8-year „Grundschule“ (former GDR before 1965)
- ☐ „Mittlere Reife“, „Mittlerer Schulabschluss“ or qualification from „Realschule“ or „Polytechnische Oberschule (POS)“
- ☐ „Fachhochschulreife“
- ☐ „Abitur“
- ☐ Other school qualification, namely: ⎿ ⏌

SDD09 What is your highest vocational qualification?

- ☐ None
- ☐ „beruflich-betriebliche Ausbildung“ (apprenticeship with vocational school)
- ☐ „beruflich-schulische Ausbildung („Berufsfachschule“, „Handelsschule“)
- ☐ „Fachschule“, „Meisterschule“, „Technikerschule“, „Berufsakademie“ or „Fachakademie“
- ☐ „Fachhochschule“ (Bachelor, Master, „Staatsexamen“, diploma; incl. „Hochschulen für angewandte Wissenschaften“)
- ☐ University (Bachelor, Master, „Magister“, „Staatsexamen“, diploma)
- ☐ Other vocational qualification, namely: ⌊ ⌋

SDD11 In which country were you born?
Please take into account the national borders at that time.

- ☐ in Germany
- ☐ in another country, namely ⌊ ⌋

SDD12 In which country were your parents born?
Please take into account the national borders at that time.

Mother

- ☐ in Germany
- ☐ in another country, namely ⌊ ⌋

Father

- ☐ in Germany
- ☐ in another country, namely ⌊ ⌋
