# Supplementary material for: Association between psychosocial factors and adverse effects of light-to-moderate ambient heat in patients with chronic diseases: results of the prospective cohort study CLIMATE-II
Source: BMC Med. 2026 Jan 15;24:52. doi: 10.1186/s12916-026-04622-4 (PMC12849320; doi:10.1186/s12916-026-04622-4)
Supplement: Supplementary file 3 — Additional file 3: Supplementary figures and tables. Figure S1: Number of participants by number of observations. Figure S2: Distribution of dependent variable and continuous independent variables. Figure S3: Mean maximum daily heat exposure in °C between 1 July and 19 September 2024 by weather measuring station. Figure S4: Number of observations by heat exposure. Table S1: Pairwise correlations between independent variables. Table S2: Unadjusted association of symptom burden with heat and participant characteristics by distance between meteorological station and study participants’ homes. Table S3: Multivariable association of symptom burden with heat and participant characteristics by distance between meteorological station and study participants’ homes. Table S4: Association of log-transformed symptom burden with heat and participant characteristics. [file 12916_2026_4622_MOESM3_ESM.pdf]

ASSOCIATION BETWEEN PSYCHOSOCIAL FACTORS AND ADVERSE EFFECTS OF LIGHT-TO-MODERATE AMBIENT HEAT IN PATIENTS WITH CHRONIC DISEASES: RESULTS OF THE PROSPECTIVE COHORT STUDY CLIMATE-II.

Additional file 3: Supplementary figures and tables

Ingmar Schäfer, Valentina Paucke, Julia Nothacker, Agata Menzel, Susanne Döpfmer, Klaus Hager, Susann Hueber, Arian Karimzadeh, Thomas Kötter, Christin Löffler, Beate S. Müller, Martin Scherer, Dagmar Lühmann

Figure S1: Number of participants by number of observations

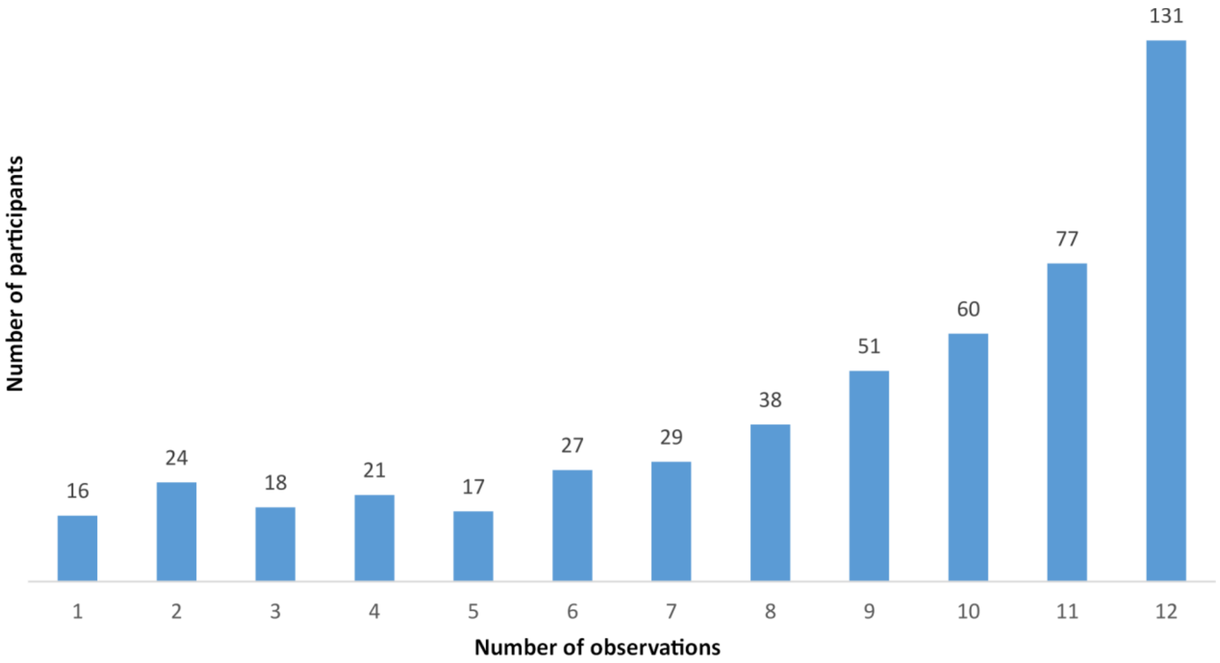

Figure S2: Distribution of dependent variable and continuous independent variables

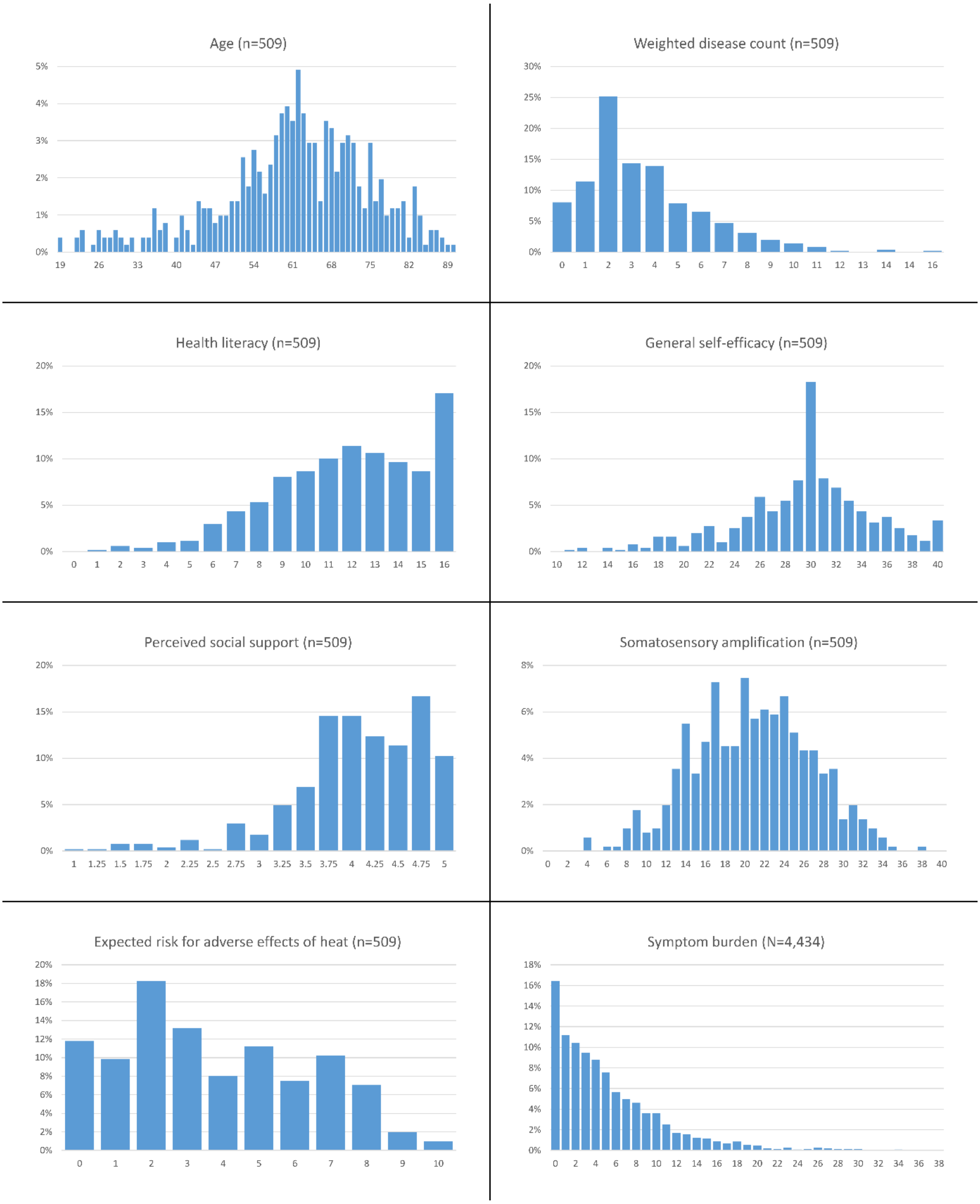

Table S1: Pairwise correlations between independent variables (n=509)

	Age	Sex: women	Living arrangement: living alone	Education: secondary	Education: primary or below	Participant born in Germany, at least one parent abroad	Participant born abroad	Weighted disease count	Health literacy	General self-efficacy	Perceived social support	Somatosensory amplification	Expected risk for adverse effects of heat
Age	1.00												
Sex: women	-0.16 P<0.001	1.00											
Living arrangement: living alone	0.04 P=0.044	0.11 P<0.001	1.00										
Education: secondary	-0.22 P<0.001	0.14 P<0.001	0.10 P<0.001	1.00									
Education: primary or below	0.12 P<0.001	-0.14 P<0.001	0.00 P=0.803	-0.69 P<0.001	1.00								
Participant born in Germany, at least one parent abroad	-0.05 P=0.004	-0.01 P=0.519	-0.02 P=0.280	0.09 P<0.001	-0.08 P<0.001	1.00							
Participant born abroad	-0.01 P=0.437	-0.06 P=0.001	-0.06 P=0.002	-0.06 P=0.001	0.05 P=0.004	-0.04 P=0.014	1.00						
Weighted disease count	0.16 P<0.001	-0.12 P<0.001	0.16 P<0.001	0.01 P=0.533	-0.08 P<0.001	0.02 P=0.229	0.04 P=0.031	1.00					
Health literacy	0.07 P<0.001	-0.05 P=0.007	-0.01 P=0.746	-0.01 P=0.430	0.14 P<0.001	0.04 P=0.034	0.08 P<0.001	-0.15 P<0.001	1.00				
General self-efficacy	0.23 P<0.001	-0.15 P<0.001	-0.13 P<0.001	-0.07 P<0.001	0.03 P=0.125	0.00 P=0.817	0.06 P=0.001	-0.13 P<0.001	0.40 P<0.001	1.00			
Perceived social support	0.01 P=0.665	0.06 P=0.002	-0.31 P<0.001	-0.05 P=0.004	0.02 P=0.286	0.04 P=0.026	0.05 P=0.005	-0.16 P<0.001	0.36 P<0.001	0.37 P<0.001	1.00		
Somatosensory amplification	0.00 P=0.806	0.30 P<0.001	-0.01 P=0.668	0.03 P=0.159	-0.08 P<0.001	-0.03 P=0.163	0.05 P=0.012	0.17 P<0.001	-0.19 P<0.001	-0.24 P<0.001	-0.10 P<0.001	1.00	
Expected risk for adverse effects of heat	-0.09 P<0.001	0.11 P<0.001	0.00 P=0.797	0.05 P=0.008	-0.09 P<0.001	0.03 P=0.114	0.06 P=0.002	0.26 P<0.001	-0.15 P<0.001	-0.18 P<0.001	-0.11 P<0.001	0.38 P<0.001	1.00

Figure S3: Mean maximum daily heat exposure in °C between 1 July and 19 September 2024 by meteorological station

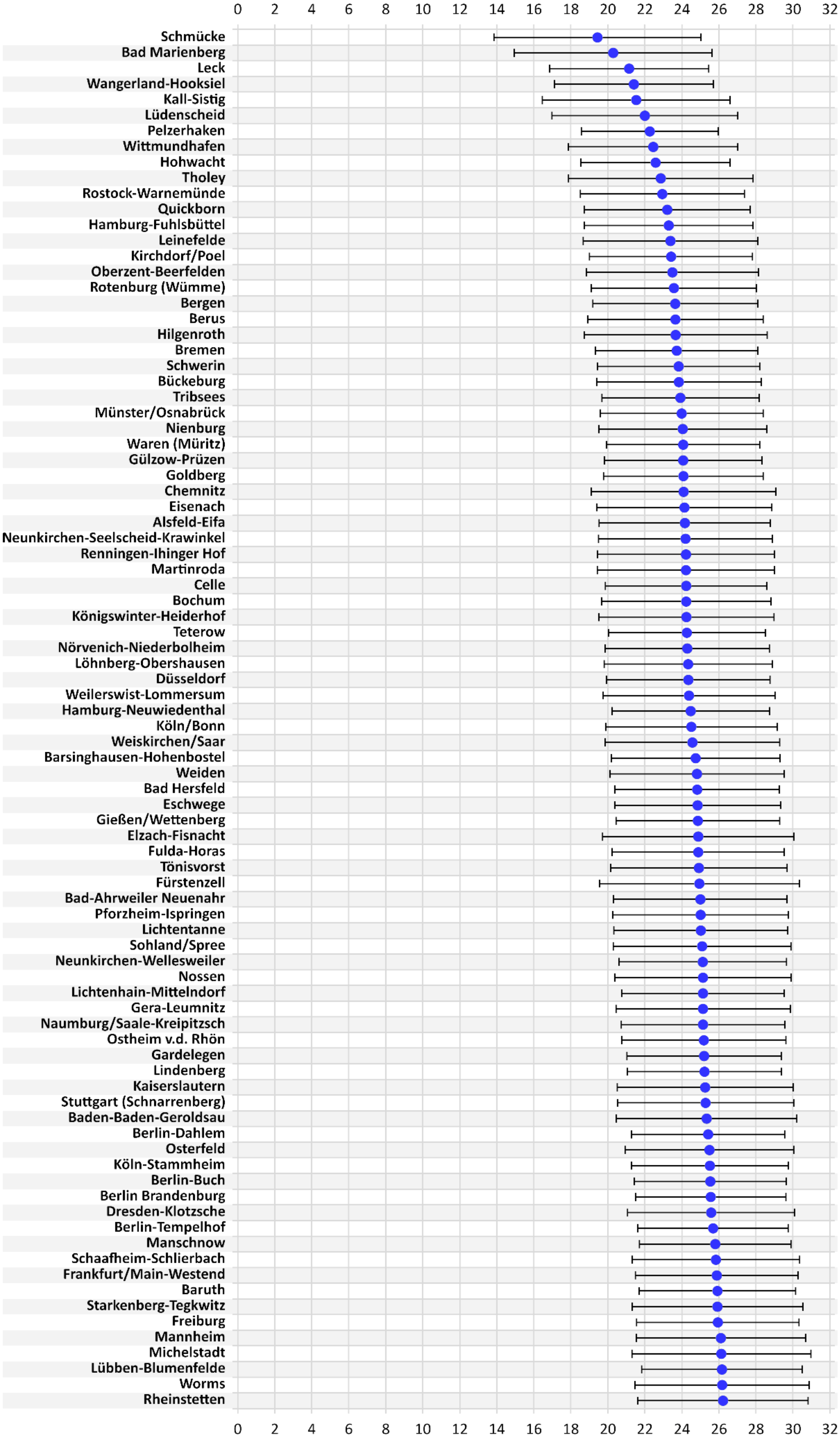

Figure S4: Number of observations by heat exposure

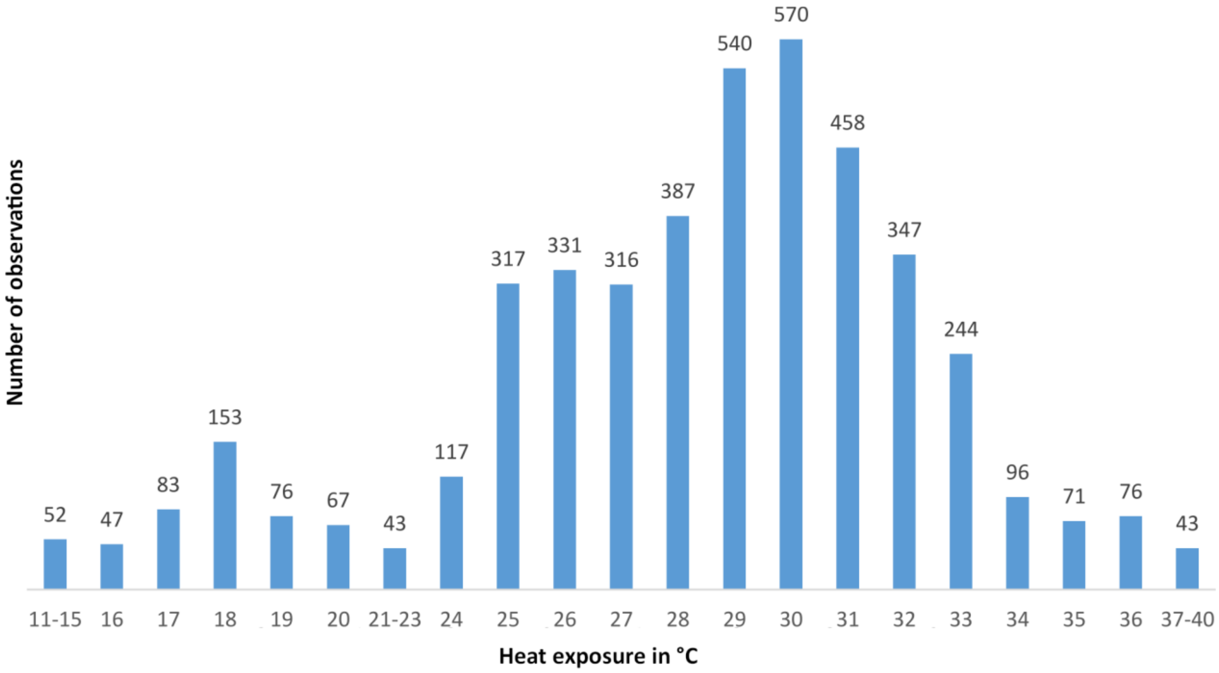

Table S2: Unadjusted association of symptom burden with heat and participant characteristics by distance between meteorological station and study participants' homes: results of linear regression analyses controlled for observation numbers and heat exposure and adjusted for random effects on the levels of meteorological stations and participants.

	Distance ≤ 11 km between station and participant (n=271; N=2,310)		Distance > 11 km between station and participant (n=257; N=2,124)	
**Characteristic**	**Coefficient (95% CI)**	**P**	**Coefficient (95% CI)**	**P**
Heat exposure				
- 27°C or less	reference		reference	
- more than 27°C through 32°C	0.95 (0.68/1.21)	<0.001	1.13 (0.83/1.43)	<0.001
- more than 32°C through 40°C	1.28 (0.93/1.63)	<0.001	1.73 (1.25/2.21)	<0.001
Age	-0.02 (-0.06/0.02)	0.267	-0.04 (-0.09/0.006)	0.091
Sex				
- men	reference		reference	
- women	1.22 (0.25/2.20)	0.014	1.57 (0.33/2.80)	0.013
Living arrangement				
- living together with others	reference		reference	
- living alone	1.41 (0.32/2.49)	0.011	0.27 (-1.38/1.91)	0.751
Educational level				
- tertiary	reference		reference	
- secondary	1.38 (0.26/2.51)	0.016	1.71 (0.29/3.12)	0.018
- primary or below	1.48 (0.04/2.92)	0.044	2.26 (0.38/4.13)	0.018
Country of birth				
- participant and parents born in Germany	reference		reference	
- participant born in Germany, at least one parent abroad	-0.09 (-2.12/1.94)	0.932	-1.45 (-4.53/1.63)	0.357
- participant born abroad	-1.42 (-3.51/0.68)	0.185	-1.28 (-5.75/3.19)	0.357
Weighted disease count	0.60 (0.41/0.79)	<0.001	0.56 (0.34/0.78)	<0.001
Health literacy	-0.39 (-0.54/-0.24)	<0.001	-0.59 (-0.77/-0.42)	<0.001
General self-efficacy	-0.38 (-0.46/-0.29)	<0.001	-0.39 (-0.50/-0.29)	<0.001
Perceived social support	-1.89 (-2.56/-1.22)	<0.001	-2.02 (-2.80/-1.24)	<0.001
Somatosensory amplification	0.29 (0.21/0.37)	<0.001	0.41 (0.32/0.50)	<0.001
Expected risk for adverse effects of heat	0.65 (0.47/0.82)	<0.001	0.96 (0.76/1.16)	<0.001

CI: confidence interval; n: number of participants; N: number of observations.

Table S3: Multivariable association of symptom burden with heat and participant characteristics by distance between meteorological station and study participants' homes: results of linear regression analyses controlled for observation numbers and adjusted for random effects on the levels of meteorological stations and participants.

	Distance ≤ 11 km between station and participant (n=271; N=2,310)		Distance > 11 km between station and participant (n=257; N=2,124)	
**Characteristic**	**Coefficient (95% CI)**	**P**	**Coefficient (95% CI)**	**P**
Heat exposure				
- 27°C or less	reference		reference	
- more than 27°C through 32°C	0.96 (0.70/1.23)	<0.001	1.12 (0.82/1.41)	<0.001
- more than 32°C through 40°C	1.31 (0.96/1.65)	<0.001	1.72 (1.25/2.20)	<0.001
Age	-0.002 (-0.03/0.03)	0.901	-0.01 (-0.05/0.03)	0.535
Sex				
- men	reference		reference	
- women	0.23 (-0.62/1.08)	0.598	0.27 (-0.72/1.25)	0.598
Living arrangement				
- living together with others	reference		reference	
- living alone	0.14 (-0.81/1.08)	0.773	-0.47 (-1.75/0.81)	0.470
Educational level				
- tertiary	reference		reference	
- secondary	0.31 (-0.61/1.23)	0.510	1.12 (0.05/2.19)	0.040
- primary or below	0.63 (-0.57/1.83)	0.302	0.60 (-0.81/2.00)	0.408
Country of birth				
- participant and parents born in Germany	reference		reference	
- participant born in Germany, at least one parent abroad	-0.44 (-2.00/1.13)	0.583	-0.41 (-2.69/1.88)	0.726
- participant born abroad	-1.71 (-3.35/-0.06)	0.042	-2.53 (-5.79/0.72)	0.127
Weighted disease count	0.33 (0.16/0.51)	<0.001	0.30 (0.12/0.48)	0.001
Health literacy	-0.09 (-0.22/0.05)	0.215	-0.21 (-0.37/-0.05)	0.012
General self-efficacy	-0.22 (-0.31/-0.12)	<0.001	-0.19 (-0.29/-0.09)	<0.001
Perceived social support	-0.41 (-1.07/0.25)	0.224	-0.75 (-1.43/-0.07)	0.031
Somatosensory amplification	0.16 (0.09/0.23)	<0.001	0.20 (0.11/0.28)	<0.001
Expected risk for adverse effects of heat	0.30 (0.13/0.46)	<0.001	0.56 (0.37/0.75)	<0.001

CI: confidence interval; n: number of participants; N: number of observations.

Table S4: Association of log-transformed symptom burden with heat and participant characteristics: results of linear regression analyses controlled for observation numbers and adjusted for random effects on the levels of meteorological stations and participants (n=509, N=4,434).

	Unadjusted models*		Multivariable model	
**Characteristic**	**Coefficient (95% CI)**	**P**	**Coefficient (95% CI)**	**P**
Heat exposure				
- 27°C or less	reference		reference	
- more than 27°C through 32°C	0.19 (0.15/0.23)	<0.001	0.19 (0.15/0.23)	<0.001
- more than 32°C through 40°C	0.26 (0.21/0.32)	<0.001	0.27 (0.21/0.32)	<0.001
Age	-0.006 (-0.01/-0.0007)	0.026	-0.001 (-0.005/0.003)	0.587
Sex				
- men	reference		reference	
- women	0.26 (0.13/0.40)	<0.001	0.10 (-0.01/0.21)	0.081
Living arrangement				
- living together with others	reference		reference	
- living alone	0.16 (-0.002/0.316)	0.053	-0.01 (-0.14/0.12)	0.879
Educational level				
- tertiary	reference		reference	
- secondary	0.26 (0.11/0.41)	0.001	0.08 (-0.04/0.20)	0.169
- primary or below	0.26 (0.06/0.46)	0.011	0.06 (-0.10/0.21)	0.460
Country of birth				
- participant and parents born in Germany	reference		reference	
- participant born in Germany, at least one parent abroad	-0.12 (-0.42/0.18)	0.432	-0.07 (-0.30/0.15)	0.507
- participant born abroad	-0.24 (-0.59/0.10)	0.165	-0.30 (-0.56/-0.04)	0.023
Weighted disease count	0.09 (0.06/0.11)	<0.001	0.04 (0.02/0.06)	<0.001
Health literacy	-0.08 (-0.10/-0.06)	<0.001	-0.03 (-0.04/-0.008)	0.004
General self-efficacy	-0.06 (-0.07/-0.05)	<0.001	-0.02 (-0.04/-0.01)	<0.001
Perceived social support	-0.32 (-0.41/-0.23)	<0.001	-0.12 (-0.20/-0.04)	0.004
Somatosensory amplification	0.06 (0.05/0.07)	<0.001	0.04 (0.03/0.04)	<0.001
Expected risk for adverse effects of heat	0.14 (0.12/0.16)	<0.001	0.08 (0.06/0.10)	<0.001

*** controlled for heat exposure;**
CI: confidence interval; n: number of participants; N: number of observations.
