# Supplementary material for: Association between psychosocial factors and adverse effects of light-to-moderate ambient heat in patients with chronic diseases: results of the prospective cohort study CLIMATE-II
Source: BMC Med. 2026 Jan 15;24:52. doi: 10.1186/s12916-026-04622-4 (PMC12849320; doi:10.1186/s12916-026-04622-4)
Supplement: Supplementary file 4 — Additional file 4. [file 12916_2026_4622_MOESM4_ESM.pdf]

STROBE Statement—Checklist of items that should be included in reports of ***cohort studies***

	Item No	Recommendation	Location
**Title and abstract**	1	(*a*) Indicate the study's design with a commonly used term in the title or the abstract	Title, pg. 1, ln. 1-2; Abstract, pg.2, ln. 40.
		(*b*) Provide in the abstract an informative and balanced summary of what was done and what was found	Abstract, pg. 2, ln. 40-56.
**Introduction**			
Background/rationale	2	Explain the scientific background and rationale for the investigation being reported	Introduction, pg. 3, ln. 67-102.
Objectives	3	State specific objectives, including any prespecified hypotheses	Introduction, pg. 4, ln. 103-110.
**Methods**			
Study design	4	Present key elements of study design early in the paper	Methods, pg. 4, ln. 113-115.
Setting	5	Describe the setting, locations, and relevant dates, including periods of recruitment, exposure, follow-up, and data collection	Methods/Participant recruitment, pg. 4, ln. 122-124; Methods/Data collection, pg. 5, ln. 153 & 167-168
Participants	6	(*a*) Give the eligibility criteria, and the sources and methods of selection of participants. Describe methods of follow-up	Methods/Participant recruitment, pg. 4-5, ln. 122-142; Methods/Data collection, pg. 5, ln. 145-166.
		(*b*) For matched studies, give matching criteria and number of exposed and unexposed	not applicable
Variables	7	Clearly define all outcomes, exposures, predictors, potential confounders, and effect modifiers. Give diagnostic criteria, if applicable	Methods/Dependent variable, pg. 5, ln. 171-176; Methods/Heat exposure, pg. 6, ln. 179-195; Methods/Other independent variables, pg. 6-7, ln. 198-261.
Data sources/ measurement	8*	For each variable of interest, give sources of data and details of methods of assessment (measurement). Describe comparability of assessment methods if there is more than one group	Methods/Data collection, pg. 5, ln. 145-166; Methods/Dependent variable, pg. 5, ln. 171-172; Methods/Heat exposure, pg. 6, ln. 184-185; Methods/Other independent variables, pg. 6-7, ln. 198, 204 & 250.
Bias	9	Describe any efforts to address potential sources of bias	Methods/Statistical analyses,

			pg. 8, ln. 265-266, 268-271, 283-287. Discussion/Strengths and limitations, pg. 14, ln. 485-492.
Study size	10	Explain how the study size was arrived at	Methods/Participant recruitment, pg. 4-5, ln. 137-141; Methods/Data collection, pg. 5, ln. 150-152 & 165-166.
Quantitative variables	11	Explain how quantitative variables were handled in the analyses. If applicable, describe which groupings were chosen and why	Methods/Statistical analyses, pg. 8, ln. 273-282.
Statistical methods	12	(*a*) Describe all statistical methods, including those used to control for confounding	Methods/Statistical analyses, pg. 8, ln. 264-282.
		(*b*) Describe any methods used to examine subgroups and interactions	Methods/Statistical analyses, pg. 8, ln. 283-287.
		(*c*) Explain how missing data were addressed	Methods/Statistical analyses, pg. 8, ln. 288.
		(*d*) If applicable, explain how loss to follow-up was addressed	Methods/Statistical analyses, pg. 8, ln. 288.
		(*e*) Describe any sensitivity analyses	Methods/Statistical analyses, pg. 8, ln. 283-287.
**Results**			
Participants	13*	(a) Report numbers of individuals at each stage of study—eg numbers potentially eligible, examined for eligibility, confirmed eligible, included in the study, completing follow-up, and analysed	Figure 1, pg. 23; Figure 2, pg. 24; Results/Recruitment, pg. 8-9, ln. 294-308.
		(b) Give reasons for non-participation at each stage	Figure 1, pg. 23.
		(c) Consider use of a flow diagram	Figure 1, pg. 23.
Descriptive data	14*	(a) Give characteristics of study participants (eg demographic, clinical, social) and information on exposures and potential confounders	Table 1, pg. 27; Table 2, pg. 28, Results/Baseline data, pg. 9, ln. 311-331. Figure S2, Supplement pg. 3.
		(b) Indicate number of participants with missing data for each variable of interest	Discussion/Strengths and limitations, pg. 14, ln. 488-489.
		(c) Summarise follow-up time (eg, average and total amount)	Results/Recruitment, pg. 9, ln. 296-300;
Outcome data	15*	Report numbers of outcome events or summary measures over time	Figure S1, Supplement pg. 2;

			Figure S4, Supplemet pg. 5; Results/Recruitment, pg. 297-300; Results/Follow-up data, pg. 336-340.
Main results	16	(*a*) Give unadjusted estimates and, if applicable, confounder-adjusted estimates and their precision (eg, 95% confidence interval). Make clear which confounders were adjusted for and why they were included	Table 3, pg. 29; Results/Unadjusted analyses, pg. 10, ln. 356-369; Results/Multivariable model, pg. 11, ln. 372-386.
		(*b*) Report category boundaries when continuous variables were categorized	Table 3, pg. 29; Methods/Heat exposure, pg. 6, ln. 190-195. Results/Unadjusted analyses, pg. 10, ln. 356-369; Results/Multivariable model, pg. 11, ln. 372-386.
		(*c*) If relevant, consider translating estimates of relative risk into absolute risk for a meaningful time period	not applicable
Other analyses	17	Report other analyses done—eg analyses of subgroups and interactions, and sensitivity analyses	Table S1, Supplement, pg. 4; Table S2, Supplement, pg. 7; Table S3, Supplement, pg. 8; Table S4, Supplement, pg. 9; Results/Baseline data, pg. 9, ln. 326-331; Results/Sensitivity analyses, pg. 11-12, ln. 389-407.
**Discussion**			
Key results	18	Summarise key results with reference to study objectives	Discussion, pg. 11, ln. 410-414.
Limitations	19	Discuss limitations of the study, taking into account sources of potential bias or imprecision. Discuss both direction and magnitude of any potential bias	Discussion/Strengths and limitations, pg. 13-14, ln. 454-494.
Interpretation	20	Give a cautious overall interpretation of results considering objectives, limitations, multiplicity of analyses, results from similar studies, and other relevant evidence	Discussion, pg. 11, ln. 410-414.

Generalisability	21	Discuss the generalisability (external validity) of the study results	Discussion/Strengths and limitations, pg. 13, ln. 470-477.
**Other information**			
Funding	22	Give the source of funding and the role of the funders for the present study and, if applicable, for the original study on which the present article is based	Funding, pg. 15, ln. 530-532.

*Give information separately for exposed and unexposed groups.

Note: An Explanation and Elaboration article discusses each checklist item and gives methodological background and published examples of transparent reporting. The STROBE checklist is best used in conjunction with this article (freely available on the Web sites of PLoS Medicine at http://www.plosmedicine.org/, Annals of Internal Medicine at http://www.annals.org/, and Epidemiology at http://www.epidem.com/). Information on the STROBE Initiative is available at http://www.strobe-statement.org.
